# A Pro-Drug Approach for Selective Modulation of AI-2-Mediated Bacterial Cell-to-Cell Communication

**DOI:** 10.3390/s120303762

**Published:** 2012-03-21

**Authors:** Min Guo, Sonja Gamby, Shizuka Nakayama, Jacqueline Smith, Herman O. Sintim

**Affiliations:** Department of Chemistry and Biochemistry, University of Maryland, Building 091, College Park, MD 20742, USA; E-Mails: mguo@umd.edu (M.G.); sonja.gamby@gmail.com (S.G.); snakayam@umd.edu (S.N.); smithjacqueline83@gmail.com (J.S.)

**Keywords:** AI-2, DPD analog, quorum sensing inhibitor, ester pro-drug, *lsr* expression, autoinducer cell permeation, autoinducer processing, enteric bacteria

## Abstract

The universal quorum sensing autoinducer, AI-2, is utilized by several bacteria. Analogs of AI-2 have the potential to modulate bacterial behavior. Selectively quenching the communication of a few bacteria, in the presence of several others in an ecosystem, using analogs of AI-2 is non-trivial due to the ubiquity of AI-2 processing receptors in many bacteria that co-exist. Herein, we demonstrate that when an AI-2 analog, isobutyl DPD (which has been previously shown to be a quorum sensing, QS, quencher in both *Escherichia coli* and *Salmonella typhimurium*) is modified with ester groups, which get hydrolyzed once inside the bacterial cells, only QS in *E. coli*, but not in *S. typhimurium*, is inhibited. The origin of this differential QS inhibition could be due to differences in analog permeation of the bacterial membranes or ester hydrolysis rates. Such differences could be utilized to selectively target QS in specific bacteria amongst a consortium of other species that also use AI-2 signaling.

## 1. Introduction

Bacteria can exist as single entities as well as be part of a community of other bacteria (which could consist of same or different species of bacteria). In either lifestyle (free flowing or community), bacteria communicate with their neighbors via small molecules called autoinducers (a process called quorum sensing, QS) [1]. It is now appreciated that QS controls the expression of virulence factors [2] or biofilm-associated genes [3,4] in a variety of clinically important bacteria. Consequently, interests in identifying the small molecules that are implicated in bacterial communication as well as the receptor proteins that are involved in the quorum sensing process have intensified [5–10]. It has been assumed that strategies that target quorum-sensing processes and not viability of bacteria should lead to less pressure for bacteria to evolve resistance mechanism, although this assumption has not yet been clinically proven. There are several instances in nature whereby some bacteria develop strategies to quench the communication of other bacteria in order to gain some competitive advantage. For example, the production of homoserine lactonases or acylases by *Bacillus* or *Pseudomonas* has been shown to lead to the destruction of autoinducers (homoserine lactones) produced by competing bacteria [11]. Also, small molecules or autoinducers produced by some bacteria or hosts have been shown to disrupt the quorum sensing of others [12–14].

Several groups have been interested in the development of small molecules that could be used to inhibit autoinducer signaling in a variety of bacteria [15–22]. Most of these small molecules have been derivatives of the natural quorum sensing molecules. For example Sugar [22], Blackwell [23] and others [24,25] have demonstrated that modifications of the species specific homoserine autoinducer, called AI-1, afford analogs that could compete with the native signaling molecule. The Janda [19,26,27] and the Sintim [20,28] groups have focused on analogs of the universal quorum sensing molecule, AI-2. AI-2 is termed universal because it is either produced or sensed by over seventy different bacterial species. Analogs of AI-2 have been shown to either act as synergistic agonists in some *Vibrio* species [26,28] or antagonist [20] in enteric bacteria, such as *E. coli* and *S. typhimurium*. Interestingly, it has been demonstrated that the nature of the C1 acyl group in AI-2 analogs confers specificity in disrupting QS processes in a variety of bacteria [20]. For example, hexyl-DPD (an analog of AI-2, which has the C1 methyl group in the native compound replaced by a hexyl group) inhibits AI-2-mediated *lsr* expression in *E. coli* whereas this same molecule is ineffective against AI-2-mediated *lsr* expression in the analogous enteric bacteria, *S. typhimurium*. On the other hand, isobutyl DPD (for this analog, the methyl group is replaced with the isobutyl group) could inhibit AI-2-mediated *lsr* expression in *S. typhimurium*, implying that subtle differences in the C1 substituents of AI-2 could result in significant differences in biological response [20].

**Figure 1.**
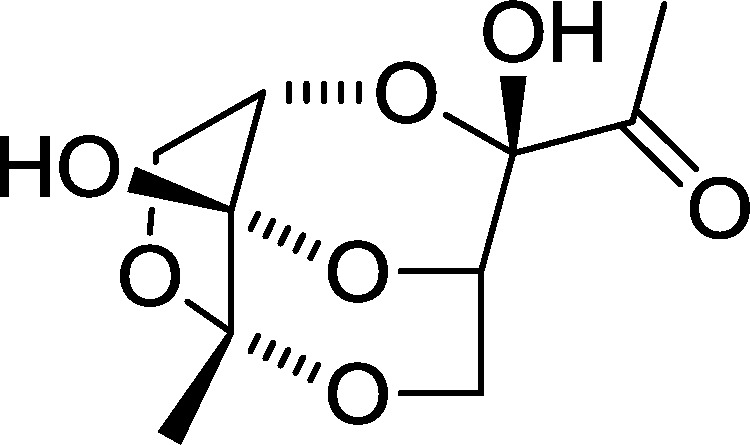
Structure of AI-2 dimer.

One of the limitations of the use of AI-2 analogs in selectively modulating bacterial behavior is the instability of these analogs. At high concentrations, it has been shown that AI-2 form dimers (see Figure 1), which are not biologically active [29]. This makes the purification of AI-2 or analogs on silica gel problematic and most studies that use synthetic AI-2 use unpurified molecules. Others have attempted to solve the instability issue associated with AI-2 by making ester derivatives that hydrolyze *in vivo* to release active autoinducers [30]. This strategy is promising in delivering purer and more stable AI-2 analogs that could be used in studying bacterial communication, with implications for disease control or synthetic biology applications. However, detailed study that correlates the nature of the ester group on AI-2 and biological activity has not been described. Additionally, as analogs of AI-2 are emerging as potent anti-QS molecules [20], it is of interest to investigate if these QS signaling inhibitors could also be protected as ester “pro-drugs” and still retain their inhibitory activity. If different bacteria processed ester-protected AI-2 analogs differently, then one could selectively modulate the activity of specific bacteria in an ecosystem via the use of differently protected AI-2 analog.

## 2. Experimental Section

### 2.1. Synthesis of Diazocarbonyls

#### 2.1.1. Generation of Diazomethane

Diazomethane was generated from Diazald^®^ (Sigma-Aldrich, St. Louis, MO, USA) using a diazomethane generator apparatus (Sigma-Aldrich, Oberkochen, Germany), following the protocol provided by Sigma-Aldrich (Oberkochen, Germany). Briefly, a solution of Diazald^®^ (5 g) in diethyl ether (45 mL) was slowly added to a solution of KOH (5 g) in mixed solvent (water (8 mL) and ethanol (10 mL)) at 65 °C over 20 min. The generated diazomethane and the diethyl ether solvent distilled and was trapped in a collecting vessel using a dry ice/isopropanol bath to give diazomethane as a solution in diethyl ether (ca. 0.4–0.5 M).

#### 2.1.2. Addition of Diazomethane to Acyl Chlorides

To a solution of diazomethane (3 equiv.) in diethyl ether was added an acyl chloride (1 equiv.) dropwise at 0 °C. The resulting solution was allowed to stir for another 2 h and warmed up gradually to room temperature. The solvent was removed under vacuum and the diazocarbonyl residue (a yellow liquid) was used for the next step without further purification.

### 2.2. Synthesis of Diazodiols

DBU (0.16–0.20 equiv.) and 2-(*tert*-butyldimethylsilyloxy) acetaldehyde (1–1.5 equiv.) were added to a solution of the crude diazocarbonyl (1 equiv.) in anhydrous acetonitrile (0.2 M). The reaction was stirred at room temperature under nitrogen for 4–8 h and monitored by TLC. Upon disappearance of starting material, the reaction was quenched with sodium bicarbonate. The organic layer was extracted with dichloromethane (3 × 20 mL) and dried with magnesium sulfate. The solvent was evaporated under reduced pressure. To a solution of crude product in anhydrous tetrahydrofuran at 0 °C, TBAF was added (1–2 equiv.). The solution was allowed to warm to room temperature and stirred for 1–3 h under nitrogen. The solvent was evaporated, and the crude product was purified by column chromatography. The products eluted as yellow oils using 1:3 to 3:2 ethyl acetate/hexane as the mobile phase.

### 2.3. Synthesis of Ester Protected Diazo Compounds

To a stirring solution of diazodiol (1 equiv.) catalytic 4-dimethylaminopyridine (DMAP) and suspended 4 Å molecular sieves in dichloromethane (DCM) was added the requisite anhydride. The reaction was allowed to gently stir at room temperature for 2–4 h until complete disappearance of starting material was indicated by TLC. The crude reaction mixture was filtered washed with saturated aqueous NaHCO_3_ solution and the organic phase was extracted with more DCM. The combined organic phases were dried with anhydrous MgSO_4_ and the solvent was evaporated at reduced pressure. The crude product was purified by column chromatography. The products eluted as yellow oils using 1:3 to 1:2 ethyl acetate/hexane as the mobile phase.

### 2.4. Synthesis of DPDs

Dimethyldioxirane in acetone (15–20 mL) was added dropwise to a solution of ester protected diazodiol (1 equiv.) in acetone (1–2 mL). The reaction was allowed to stir at room temperature (1–2 h) until complete disappearance of starting material was indicated by TLC (loss of UV activity). Solvent and excess reagents were evaporated under reduced pressure.

### 2.5. Bacterial Strains and Growth Conditions

Table 1 lists the bacterial strains used in this study. *S. typhimurium* and *E. coli* strains were cultured in Luria-Bertani medium (LB, Sigma, St. Louis, MO, USA). These antibiotics were used for the following strains: (60 μg·mL^−1^) kanamycin for *S. typhimurium* (MET715) and (50 μg·mL^−1^) ampicillin for *E. coli* (LW7).

### 2.6. Measurement of the QS Response (lsr Expression)

The QS response indicated by *lsr* gene expression was analyzed in pure culture studies by culturing *E. coli* LW7 pLW11 and *S. typhimurium* MET715 overnight at 30 °C in LB medium supplemented with appropriate antibiotics as stated previously. These cells were then diluted into fresh LB medium (with antibiotics) and grown to an OD_600_ of 0.4–0.8 at 37 °C, 250 rpm. Cells were then collected by centrifugation at 10,000× *g* for 10 min and resuspended in 10 mM phosphate buffer. AI-2 (20 μM) and the respective analog (20 μM) were added to the *E. coli* or *S. typhimurium* suspension for 2 h at 37 °C. AI-2 dependent β-galactosidase production was quantified by the Miller assay.

## 3. Results and Discussion

**Scheme 1.**
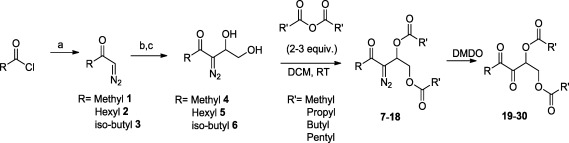
Synthetic strategy for making *bis*-ester protected AI-2 analogs. Reagents and conditions: (**a**) diazomethane, 0 °C; (**b**) *tert*-butyl-siloxyacetaldehyde, DBU (1,8 diazabicycloundec-7-ene), CH_3_CN, RT; (**c**) TBAF/THF. DCM = dichloromethane; DMDO = dimethyldioxirane.

The syntheses of *bis*-ester protected AI-2 and analogs **19**–**30** were achieved via the strategy shown in Scheme 1 [20,28]. Briefly, an aldol reaction between diazocarbonyls **1**–**3** and 2-(*tert*-butyldimethylsilyloxy) acetaldehyde afforded diazodiols **4**–**6**, after deprotection of the TBS group with TBAF. Oxidation of the diazo group in diazodiols **4**–**6** afforded AI-2 or analogs but for the production of ester protected AI-2 and analogs, it was important to perform the esterification step first to give *bis*-ester **7**–**18** before the oxidation of the diazo *bis*-ester to give targeted compounds **19**–**30**.

**Figure 2.**
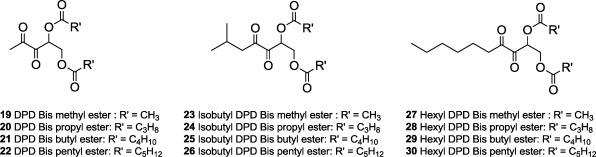
Compounds evaluated as *bis*-ester protected AI-2 analogs.

With the various AI-2 or analog ester derivatives (methyl to pentyl esters, Figure 2) in hand, we proceeded to investigate the biological profiles of these esters. We have previously demonstrated that AI-2 analogs with longer C1-acyl chains permeate more readily into bacterial cells than shorter chains [31]. This is presumably due to the favorable interactions of the alkyl chain with the phospholipid of the bacterial membrane. Based on this earlier work, we hypothesized that the longer chain ester derivatives (such as butyl or pentyl) would permeate more readily into bacterial cells than the shorter chain analogs, such as the methyl ester series [31]. However, if the cellular esterases were sensitive to the size of the esters, then the longer chain analogs would be hydrolyzed slower than the shorter chain ones. Because biological activity of ester prodrugs is dependent on permeation and prodrug activation and both of these processes would depend on the organism in question, it is not always easy to predict *a priori* which ester group is most suitable for derivatizing biologically active molecules.

**Figure 3.**
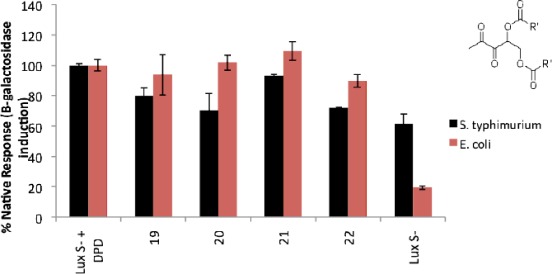
Black bars: AI-2 or analogs-mediated expression of β-galactosidase in *S. typhimurium* (MET715: LuxS^−^). Pink bars: AI-2 or analogs-mediated expression of β-galactosidase in *E. coli* LW7/LuxS^−^. AI-2 or *bis*-ester analogs of AI-2 (20 μM) were added to the bacterial strains, which do not produce their own AI-2. Compounds **19**–**22** represent ester-protected DPD analogs: **19**: DPD *bis-*methyl ester; **20**: DPD *bis-*propyl ester; **21**: DPD *bis-*butyl ester; **22**: DPD *bis-*pentyl ester.

*Bis*-ester-protected AI-2 analogs (with different ester chains; methyl, propyl, butyl and pentyl) were all effective *lsr* expression inducers in *E. coli* (see Figure 3). For *S. typhimurium*, it appears that LsrR is not as good a repressor (compared to *E. coli*) and significant expression of the *lacZ* gene was observed even in the absence of added DPD (see control, Figure 3). Nonetheless, it is apparent that more LacZ protein was present in *S. typhimurium* in the presence of AI-2 than when AI-2 was not present [about 30% more LacZ present when AI-2 is added; see Figure 3, compare the histograms for “LuxS- + AI-2” and LuxS- (no AI-2 added)].

**Figure 4.**
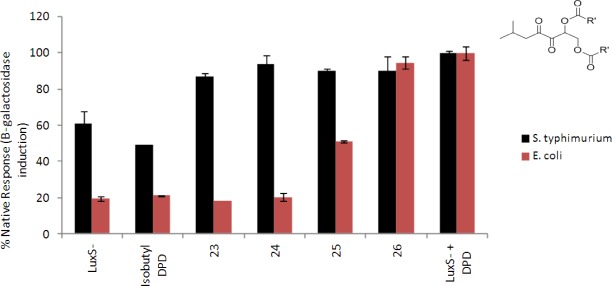
Inhibition of AI-2-mediated β-galactosidase expression in *S. typhimurium* (black bars) and *E. coli* (pink bars) with various *bis*-ester analogs of isobutyl DPD. [AI-2] = 20 μM, [analogs] = 20 μM. Compounds **23**–**26** represent ester protected isobutyl DPD analogs: **23**: isobutyl DPD *bis-*methyl ester; **24**: isobutyl DPD *bis*-propyl ester; **25**: isobutyl DPD *bis-*butyl ester; **26**: isobutyl DPD *bis-*pentyl ester.

Therefore, even if *lacZ* expression is not solely controlled by AI-2, one can safely conclude that AI-2 plays some role in *lacZ* expression in the *S. typhimurium* [32]. The origin of “leaky” *lacZ* expression in the absence of LuxS, which makes AI-2, could be due to myriads of factors, such as a lower affinity of LsrR to the LsrR DNA binding region in *S. typhimurium* (compared to *E. coli*) or a higher concentration of other phosphorylated AI-2-like molecules (such as ribulose-5-phosphate) in *S. typhimurium* (compared to *E. coli*) or lower levels of LsrR in *S. typhimurium* (compared to *E. coli*) or even non-enzymatic production of AI-2 from ribulose-5-phosphate [33]. Without experimental data to pinpoint the origin of the differential *lsr* expression in *S. typhimurium* (compared to *E. coli*), it is dangerous to make definitive statements about the origin of this difference. Despite this high LacZ background in *S. typhimurium*, we can still conclude that the majority of the ester protected DPD analogs were not as good as DPD in inducing *ls*r expression in *S. typhimurium*, and only *bis*-butyl DPD appears to be as good as DPD (see Figure 3). It is important to note that DPD/AI-2 gets into *S. typhimurium* via a ribose transporter, such as LsrB, whereas the analogs would have to diffuse into the cells, probably via passive diffusion through the membrane. Hence the marginal differences in activity observed between *bis*-butyl DPD and the other ester-protected DPD could be due to differences in membrane transport. Next, we investigated the antagonistic profile of the *bis*-ester analogs of isobutyl DPD in both *E. coli* and *S. typhimurium*. Isobutyl DPD is an antagonist of AI-2-mediated QS in both *E. coli* and *S. typhimurium* and stable versions of this analog have the potential to disrupt QS processes in these enteric bacteria, which sometimes cause food-borne diseases. For this assay, AI-2 was added to a LuxS-deficient strain of *E. coli* (LW7) or *S. typhimurium* (MET715) to induce *lsr* expression via the derepression of LrsR by phospho-AI-2 [34]. In *E. coli*, *bis*-methyl and *bis*-propyl DPD analogs were as effective QS quenchers as the unprotected isobutyl DPD (see Figure 4).

**Figure 5.**
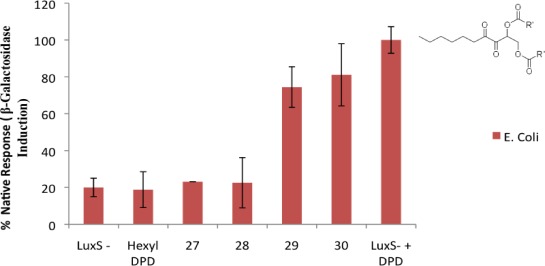
Inhibition of AI-2-mediated β-galactosidase expression in *E. coli* with various *bis*-ester analogs of hexyl DPD. [DPD] = 20 μM, [analogs] = 20 μM. Compounds **27**–**30** represent ester protected hexyl DPD analogs; **27**: hexyl DPD *bis*-methyl ester; **28**: hexyl DPD *bis*-propyl ester; **29**: hexyl DPD *bis*-butyl ester; **30**: hexyl DPD *bis*-pentyl ester.

Increasing the length of the ester chain to butyl or pentyl either reduced (butyl) or abrogated (pentyl) the inhibitory profile of the DPD analog. In *E. coli*, the same trend was also observed for the *bis*-ester derivatives of hexyl DPD (*bis*-methyl and *bis*-propyl analogs, but not butyl or pentyl derivatives, were QS inhibitors, Figure 5).

In *S. typhimurium*, however, none of the *bis*-ester protected isobutyl DPD analogs were able to antagonize the action of AI-2. Addition of isobutyl DPD to *S. typhimurium* however decreased *lacZ* expression by about 50% (compare black bar corresponding to “isobutyl DPD” to black bar corresponding to “LuxS- + DPD” in Figure 4). Thus, *S. typhimurium* and *E. coli* have similar QS systems, but differences in the processing of ester analogs of isobutyl DPD allows for the selective modulation of QS processing in *E. coli* but not in *S. typhimurium*.

## 4. Conclusions

**Figure 6.**
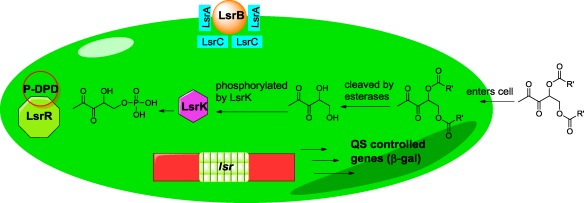
Proposed model of action in enteric bacteria. Ester protected DPD analogs diffuse into the cell, where esterases hydrolyze the ester pro-DPD and analogs and the DPD or analogs are subsequently phosphorylated by LsrK.

In conclusion, we have shown that ester derivatives of DPD analogs can be hydrolyzed inside bacterial cells to reveal the biologically active diol unit for quorum sensing disruption. We reveal that it is possible to achieve selectivity of QS modulation amongst closely related bacteria (in our case, between *E. coli* and *S. typhimurium*) via the use of ester protection of the diol unit of AI-2. The origin of this selectivity remains unknown at this moment but it could be a number of several factors, including selective permeation of the analogs or different sensitivities of the esterases required for analog hydrolysis in the different bacteria (Figure 6). Future work will be aimed at obtaining a more in depth molecular understanding of these interesting observations. This work adds to the growing list of different strategies that can be used to intercept AI-2 signaling in diverse bacteria [13,35–38].

## 



## Figures and Tables

**Table 1.** Bacterial strains used in this study.

**Strain**	**Relevant genotype and/or property**
LW7	*Escherichia coli* W3110Δ*lacU160-tna2* Δ*luxS*: Kan (LuxS-deficient: does not produce AI-2)
MET715	*Salmonella typhimurium* *rpsl putRA:* Kan*-lsr-lacZYA luxS:* T-POP (LuxS-deficient: does not produce AI-2)
