# Supplementary material for: A Pro-Drug Approach for Selective Modulation of AI-2-Mediated Bacterial Cell-to-Cell Communication
Source: Sensors (Basel). 2012 Mar 21;12(3):3762–72. doi: 10.3390/s120303762 (PMC3376627; doi:10.3390/s120303762)
Supplement: Supplementary file 1 [file sensors-12-03762-s001.pdf]

Supplementary Information

A Pro-Drug Approach for Selective Modulation of AI-2-Mediated Bacterial Cell-to-Cell Communication

Min Guo, Sonja Gamby, Shizuka Nakayama, Jacqueline Smith and Herman O. Sintim *

Department of Chemistry and Biochemistry, University of Maryland, Building 091, College Park, MD 20742, USA; E-Mails: mguo@umd.edu (M.G.); sonja.gamby@gmail.com (S.G.); snakayam@umd.edu (S.N.); smithjacqueline83@gmail.com (J.S.)

***** Author to whom correspondence should be addressed; E-Mail: hsintim@umd.edu; Tel.: +1-301-405-0633.

Received: 29 December 2011; in revised form: 26 February 2012 / Accepted: 15 March 2012 / Published: 21 March 2012

Scheme S1. Synthesis of ester protected diazodiol.

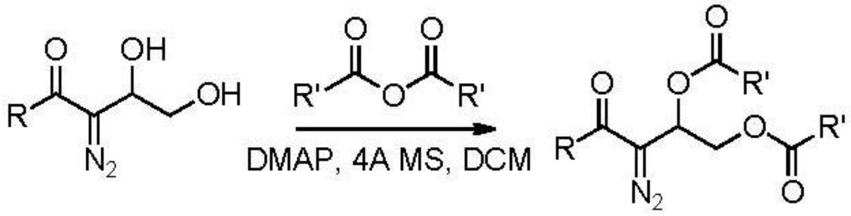

3-diazo-4-oxopentane-1,2-diyl diacetate (7): To a solution of methyl diazodiol **4**[1] (89 mg, 0.62 mmol), DAMP (15 mg, 0.2 equiv.) and suspended 4A molecular sieves (0.2 g) in anhydrous dichloromethane (DCM) was added acetic anhydride (0.12 mL, 2 equiv.). The reaction was gently stirred at room temperature overnight. The reaction was quenched with saturated aqueous $NaHCO_3$ solution. The organic and aqueous layers were separated and the organic layer was washed with brine and dried over anhydrous $MgSO_4$. The solvent was removed *in vacuo* and the residue was purified on silica gel, using 3:4 ethyl acetate/hexane as the mobile phase. The product eluted as a yellow oil (yield = 90 mg, 64%).

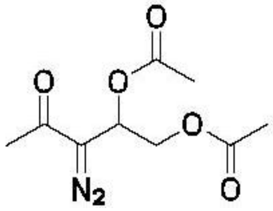

3-diazo-4-oxopentane-1,2-diyl dibutyrate (8): To a solution of methyl diazodiol **4** (23 mg, 0.16 mmol), DMAP (4 mg, 0.2 equiv.) and suspended 4A molecular sieves (0.1 g) in anhydrous dichloromethane (DCM) was added butyric anhydride (0.05 mL, 2 equiv.). The reaction was stirred at room temperature overnight. The reaction was quenched with saturated aqueous $NaHCO_3$ solution. The organic and aqueous layers were separated and the organic layer was washed with brine and dried over anhydrous $MgSO_4$. The solvent was removed *in vacuo* and the residue was purified on silica gel, using 1:5 ethyl acetate/hexane as the mobile phase. The product eluted as a yellow oil (yield = 23 mg, 51%).

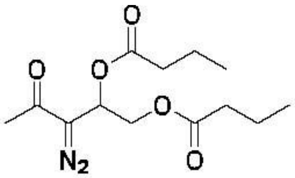

3-diazo-4-oxopentane-1,2-diyl dipentanoate (9): To a solution of methyl diazodiol **4** (23 mg, 0.16 mmol), DMAP (4 mg, 0.2 equiv.) and suspended 4A molecular sieves (0.1 g) in anhydrous dichloromethane (DCM) was added butyric anhydride (0.05 mL, 2 equiv.). The reaction was stirred at room temperature overnight. The reaction was quenched with saturated aqueous NaHCO3 solution. The organic and aqueous layers were separated and the organic layer was washed with brine and dried over anhydrous MgSO4. The solvent was removed *in vacuo* and the residue was purified on silica gel, using 1:5 ethyl acetate/hexane as the mobile phase. The product eluted as a yellow oil (yield = 27 mg, 54%).

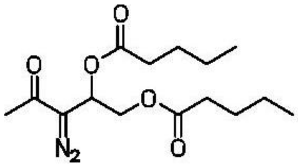

3-diazo-4-oxopentane-1,2-diyl dihexanoate (10): To a solution of methyl diazodiol **4** (23 mg, 0.16 mmol), DMAP (4 mg, 0.2 equiv.) and suspended 4A molecular sieves (0.1 g) in anhydrous dichloromethane (DCM) was added butyric anhydride (0.05 mL, 2 equiv.). The reaction was stirred at room temperature overnight. The reaction was quenched with saturated aqueous NaHCO3 solution. The organic and aqueous layers were separated and the organic layer was washed with brine and dried over anhydrous MgSO4. The solvent was removed *in vacuo* and the residue was purified on silica gel, using 1:5 ethyl acetate/hexane as the mobile phase. The product eluted as a yellow oil (yield = 38 mg, 67%).

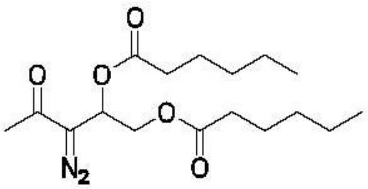

Scheme S2. Oxidation of diazo moiety into carbonyl.

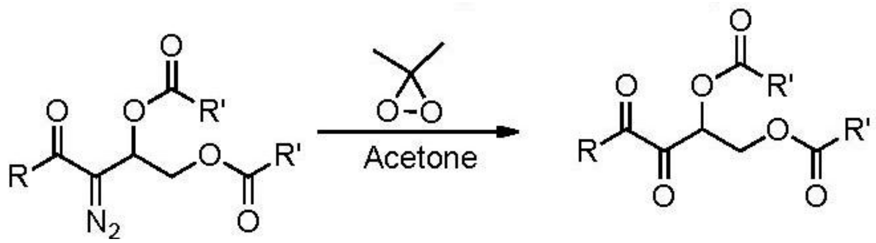

3,4-dioxopentane-1,2-diyl diacetate (19): To a solution of methyl diazo diacetate **7** (20 mg, 0.088 mmol) in acetone (1 mL) was added dimethyldioxirane acetone solution (2.5 mL, ca. 0.07–0.09 M). The resulting mixture was stirred for 1 h and solvent and excess reagents were removed under reduced pressure to obtain **19** as bright yellow oil (yield = 19 mg, quantitative).

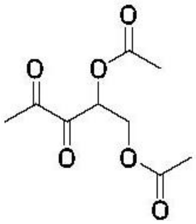

3,4-dioxopentane-1,2-diyl dibutyrate (20): To a solution of methyl diazo dibutyrate **8** (23 mg, 0.081 mmol) in acetone (1 mL) was added dimethyldioxirane acetone solution (2.5 mL, ca. 0.07-0.09 M). The resulting mixture was stirred for 1 h and solvent and excess reagents were removed under reduced pressure to obtain **20** as bright yellow oil (yield = 22 mg, quantitative).

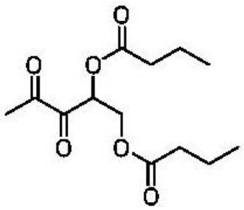

3,4-dioxopentane-1,2-diyl dipentanoate (21): To a solution of methyl diazo dipentanoate **9** (27 mg, 0.086 mmol) in acetone (1 mL) was added dimethyldioxirane acetone solution (2.5 mL, ca. 0.07–0.09 M). The resulting mixture was stirred for 1 h and solvent and excess reagents were removed under reduced pressure to obtain **21** as bright yellow oil (yield = 26 mg, quantitative).

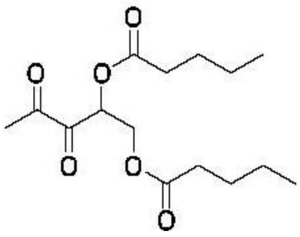

3,4-dioxopentane-1,2-diyl dihexanoate (22): To a solution of methyl diazo dihexanoate **10** (38 mg, 0.112 mmol) in acetone (1 mL) was added dimethyldioxirane acetone solution (2.5 mL, ca. 0.07–0.09 M). The resulting mixture was stirred for 1 h and solvent and excess reagents were removed under reduced pressure to obtain **22** as bright yellow oil (yield = 36.8 mg, quantitative).

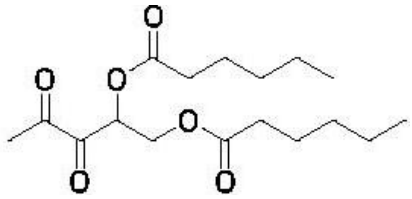

Scheme S3. NMR and MS data: NMR spectra were measured on Bruker AV-400, Bruker DRX-400 (1H at 400 MHz, ^{13}C at 100 MHz). Data for ^{1}H-NMR spectra are reported as follows: chemical shift (ppm, relative to residual solvent peaks or indicated external standards; s = singlet, t = triplets, m = multiplet), coupling constant (Hz), and integration. Data for ^{13}C-NMR are reported in terms of chemical shift (ppm) relative to residual solvent peak. Mass spectra (MS) were recorded by JEOL AccuTOF-CS (ESI positive, needle voltage 1800~2400 eV).

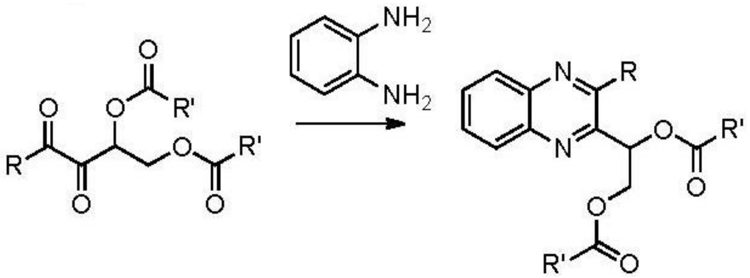

Note: Both the diazoketones and diketones did not have good MS spectrum (presumably due to decomposition under the MS conditions. Therefore, further MS characterization was done by adding phenyl diamine to the diketones and stirred overnight to convert the diketones into quinoxalines, see Scheme below (this is a standard practice). **MS data is therefore reported for the quinoxaline derivatives**.

3-diazo-4-oxopentane-1,2-diyl diacetate (7): ^{1}H NMR ($CDCl_3$, 400 MHz) δ: 5.85–5.73 (m, 1H), 4.45–4.34 (m, 1H), 4.31–4.17 (m, 1H), 2.19 (s, 3H), 2.03 (s, 3H), 2.01 (s, 3H). ^{13}C NMR ($CDCl_3$, 100 MHz) δ: 188.6, 170.1, 169.6, 68.5, 65.7, 63.5, 25.4, 20.7, 20.5.

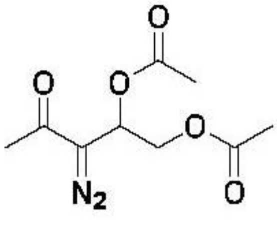

3-diazo-4-oxopentane-1,2-diyl dibutyrate (8): ^{1}H NMR ($CDCl_3$, 400 MHz) δ: 5.93–5.83 (m, 1H), 4.55–4.43 (m, 1H), 4.38–4.27 (m, 1H), 2.38–2.28 (m, 4H), 2.26 (s, 3H), 1.73–1.59 (m, 4H), 1.01–0.89 (m, 6H). ^{13}C NMR ($CDCl_3$, 100 MHz) δ: 173.3, 172.7, 65.9, 63.7, 37.0, 36.3, 36.2, 31.3, 25.9, 18.7, 14.0, 13.9.

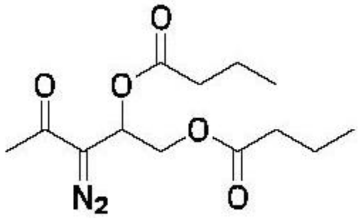

3-diazo-4-oxopentane-1,2-diyl dipentanoate (9): ^{1}H NMR ($CDCl_3$, 400 MHz) δ: 5.93–5.82 (m, 1H), 4.54–4.43 (m, 1H), 4.37–4.26 (m, 1H), 2.41–2.29 (m, 4H), 2.26 (s, 3H), 1.67–1.54 (m, 4H), 1.42–1.28 (m, 4H), 0.97–0.85 (m, 6H). ^{13}C NMR ($CDCl_3$, 100 MHz) δ: 189.0, 173.4, 172.9, 67.0, 66.0, 63.7, 34.1, 34.0, 27.2, 25.9, 22.6, 22.5, 14.0.

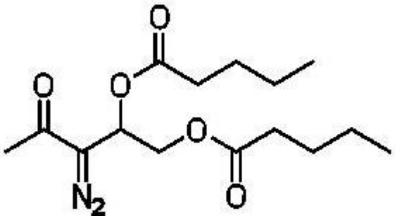

3-diazo-4-oxopentane-1,2-diyl dihexanoate (10): ^{1}H NMR ($CDCl_3$, 400 MHz) δ: 5.92–5.82 (m, 1H), 4.55–4.42 (m, 1H), 4.37–4.26 (m, 1H), 2.40–2.28 (m, 4H), 2.26 (s, 3H), 1.68–1.56 (m, 4H), 1.38–1.25 (m, 8H), 0.96–0.83 (m, 6H). ^{13}C NMR ($CDCl_3$, 100 MHz) δ: 173.4, 172.9, 66.0, 63.7, 34.4, 34.3, 31.6, 25.9, 24.9, 22.7, 14.3.

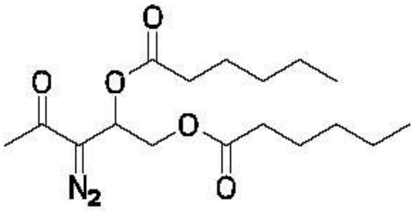

3-diazo-6-methyl-4-oxoheptane-1,2-diyl diacetate (11): ^{1}H NMR ($CDCl_3$, 400 MHz) δ: 5.85–5.76 (m, 1H), 4.44–4.34 (m, 1H), 4.32–4.21 (m, 1H), 2.28 (d, *J* = 6.7 Hz, 2H), 2.15–2.07 (m, 1H), 2.03 (s, 3H), 2.01 (s, 3H), 0.89 (d, *J* = 8.0 Hz, 6H). ^{13}C NMR ($CDCl_3$, 100 MHz) δ: 170.5, 169.9, 66.3, 63.8, 47.1, 26.0, 22.8, 22.7, 21.0, 20.8.

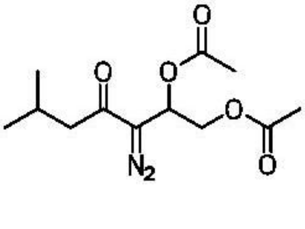

3-diazo-6-methyl-4-oxoheptane-1,2-diyl dibutyrate (12): 1H NMR (CDCl3, 400 MHz) δ: 5.92–5.81 (m, 1H), 4.50–4.39 (m, 1H), 4.34–4.22 (m, 1H), 2.37–2.21 (m, 6H), 2.18–2.04 (m, 1H), 1.69-1.54 (m, 4H), 0.96-0.85 (m, 12H). 13C NMR (CDCl3, 100 MHz) δ: 173.2, 172.6, 66.1, 63.6, 47.2, 36.2, 36.1, 26.0, 22.8, 22.7, 18.7, 18.6, 13.9.

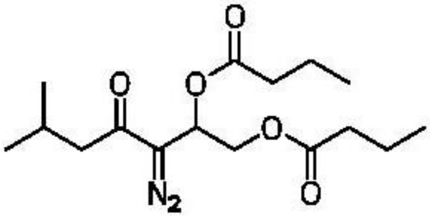

3-diazo-6-methyl-4-oxoheptane-1,2-diyl dipentanoate (13): ^{1}H NMR ($CDCl_3$, 400 MHz) δ: 5.94–5.82 (m, 1H), 4.53–4.44 (m, 1H), 4.40–4.29 (m, 1H), 2.41–2.29 (m, 6H), 2.23–2.10 (m, 1H), 1.67–1.56 (m, 4H), 1.41-1.31 (m, 4H), 0.98–0.89 (m, 12H). 13C NMR ($CDCl_3$, 100 MHz) δ: 180.0, 173.5, 172.9, 66.2, 63.7, 47.3, 34.2, 34.1, 34.0, 27.3, 22.6, 22.5, 14.1, 14.0.

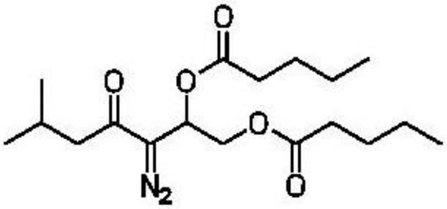

3-diazo-6-methyl-4-oxoheptane-1,2-diyl dihexanoate (14): ^{1}H NMR ($CDCl_3$, 400 MHz) δ: 5.96–5.82 (m, 1H), 4.54–4.42 (m, 1H), 4.40–4.26 (m, 1H), 2.42–2.26 (m, 6H), 2.22–2.09 (m, 1H), 1.68–1.56 (m, 4H), 1.38–1.22 (m, 8H), 1.00–0.93 (m, 6H), 0.93–0.85 (m, 6H). ^{13}C NMR ($CDCl_3$, 100 MHz) δ: 173.4, 172.8, 66.2, 63.7, 47.3, 34.3, 31.6, 24.9, 22.7, 14.3.

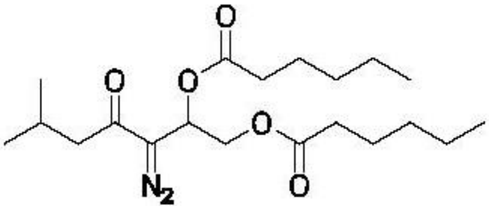

3-diazo-4-oxodecane-1,2-diyl diacetate (15): ^{1}H NMR ($CDCl_3$, 400 MHz) δ: 5.95–5.85 (m, 1H), 4.54–4.43 (m, 1H), 4.41–4.29 (m, 1H), 2.49 (t, J = 7.3 Hz, 2H), 2.12 (s, 3H), 2.11 (s, 3H), 1.69–1.60 (m, 2H), 1.38–1.27 (m, 6H), 0.91 (t, J = 6.9 Hz, 3H). ^{13}C NMR ($CDCl_3$, 100 MHz) δ: 170.7, 170.1, 66.4, 64.0, 38.6, 31.9, 29.2, 24.8, 22.9, 21.2, 21.0, 14.4.

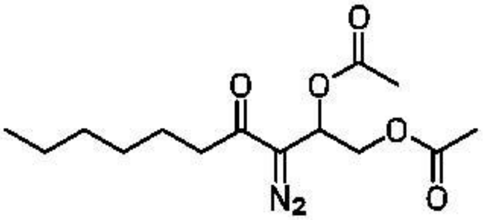

3-diazo-4-oxodecane-1,2-diyl dibutyrate (16): ^{1}H NMR (CDCl3, 400 MHz) δ: 5.96–5.85 (m, 1H), 4.56–4.46 (m, 1H), 4.40–4.28(m, 1H), 2.53–2.43 (m, 2H), 2.38–2.29 (m, 4H), 1.72–1.61 (m, 6H), 1.37–1.25 (m, 6H), 1.02–0.94 (m, 6H), 0.94–0.87 (m, 3H). ^{13}C NMR ($CDCl_3$, 100 MHz) δ: 173.3, 172.7, 66.2, 63.7, 36.3, 36.2, 31.9, 29.2, 24.9, 22.9, 18.7, 14.4, 14.0.

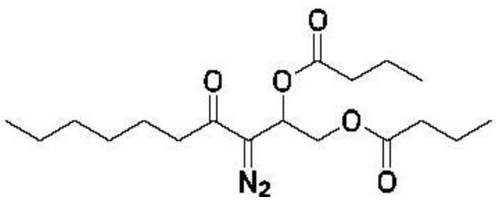

3-diazo-4-oxodecane-1,2-diyl dipentanoate (17): ^{1}H NMR ($CDCl_3$, 400 MHz) δ: 5.95–5.84 (m, 1H), 4.56–4.43 (m, 1H), 4.40–4.27 (m, 1H), 2.55–2.44 (m, 2H), 2.40–2.33 (m, 6H), 1.69–1.59 (m, 6H), 1.43–1.28 (m, 8H), 0.98–0.88 (m, 9H). ^{13}C NMR ($CDCl_3$, 100 MHz) δ: 180.7, 173.5, 172.9, 66.2, 63.7, 34.2, 34.1, 31.9, 29.2, 27.3, 27.1, 24.9, 22.9, 22.6, 14.1, 14.1.

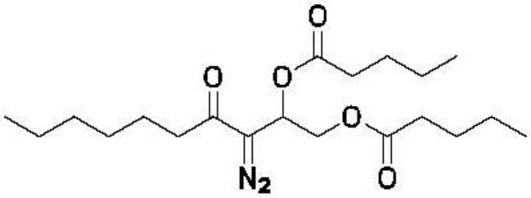

3-diazo-4-oxodecane-1,2-diyl dihexanoate (18): ^{1}H NMR ($CDCl_3$, 400 MHz) δ: 5.97–5.83 (m, 1H), 4.56–4.42 (m, 1H), 4.40–4.27 (m, 1H), 2.57–2.2.43 (m, 2H), 2.43–2.28 (m, 6H), 1.73–1.57 (m, 6H), 1.40–1.26 (m, 12H), 0.97–0.86 (m, 9H). 13C NMR ($CDCl_3$, 100 MHz) δ: 180.2, 173.5, 172.9, 66.2, 63.7, 34.4, 31.6, 24.9, 24.8, 22.9, 22.7, 14.4, 14.3.

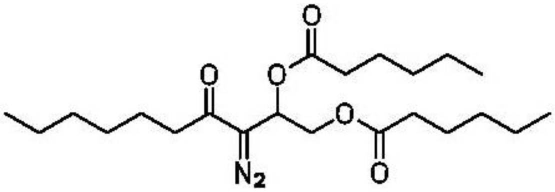

3,4-dioxopentane-1,2-diyl diacetate (19): ^{1}H NMR ($CDCl_3$, 400 MHz) δ: 5.88–5.83 (m, 1H), 4.72–4.64 (m, 1H), 4.40–4.32 (m, 1H), 2.39 (s, 3H), 2.18 (s, 3H), 2.06 (s, 3H). ^{13}C NMR ($CDCl_3$, 100 MHz) δ: 196.3, 190.4, 171.5, 170.5, 62.7, 24.1, 21.0, 20.7. Derivatization using 1,2-diaminobenzene MS (ESI): Calcd for $[C_{15}H_{16}N_2O_4 + H]^+$ 289.1188, found 289.1234.

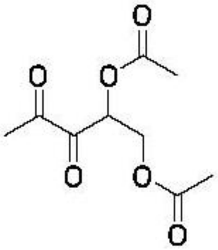

3,4-dioxopentane-1,2-diyl dibutyrate (20): ^{1}H NMR ($CDCl_3$, 400 MHz) δ: 5.90–5.84 (m, 1H), 4.75–4.66 (m, 1H), 4.41–4.32 (m, 1H), 2.46–2.40 (m, 2H), 2.39 (s, 3H), 2.29 (t, J = 7.4 Hz, 2H), 1.73–1.66 (m, 2H), 1.66–1.58 (m, 2H), 0.99 (t, J = 7.4 Hz, 3H), 0.94 (t, J = 7.4 Hz, 3H). ^{13}C NMR ($CDCl_3$, 100 MHz) δ: 196.3, 190.5, 174.1, 173.1, 62.5, 36.1, 35.8, 24.1, 18.7, 18.6, 13.9. Derivatization using 1,2-diaminobenzene MS (ESI): Calcd for $[C_{19}H_{24}N_2O_4 + H]^+$ 345.1814, found 345.1709.

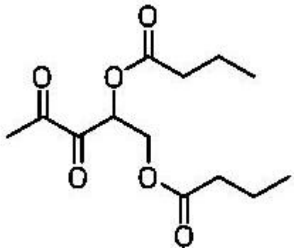

3,4-dioxopentane-1,2-diyl dipentanoate (21): ^{1}H NMR ($CDCl_3$, 400 MHz) δ: 5.89–5.83 (m, 1H), 4.74–4.65 (m, 1H), 4.41–4.32 (m, 1H), 2.48–2.41 (m, 2H), 2.40 (s, 3H), 2.34–2.28 (m, 2H), 1.70–1.61 (m, 2H), 1.61–1.53 (m, 2H), 1.44–1.30 (m, 4H), 0.98–0.89 (m, 6H). ^{13}C NMR ($CDCl_3$, 100 MHz) δ: 196.3, 190.5, 174.3, 173.3, 62.5, 34.0, 33.7, 27.2, 24.1, 22.5, 14.1, 14.0. Derivatization using 1,2-diaminobenzene MS (ESI): Calcd for $[C_{21}H_{28}N_2O_4 + H]^+$ 373.2127, found 373.2109.

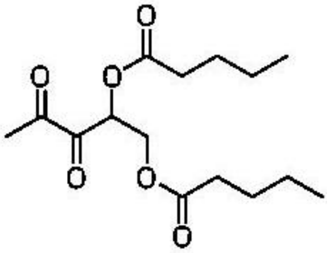

3,4-dioxopentane-1,2-diyl dihexanoate (22): ^{1}H NMR ($CDCl_3$, 400 MHz) δ: 5.91–5.82 (m, 1H), 4.75–4.64 (m, 1H), 4.42–4.30 (m, 1H), 2.47–2.40 (m, 2H), 2.39 (s, 3H), 2.33–2.25 (m, 3H), 1.72–1.62 (m, 2H), 1.62–1.53 (m, 2H), 1.40–1.21 (m, 8H), 0.97–0.83 (m, 6H). ^{13}C NMR ($CDCl_3$, 100 MHz) δ: 196.3, 190.5, 174.2, 173.3, 72.9, 62.5, 34.2, 33.9, 31.5, 24.8, 24.1, 22.7, 22.6, 14.3. Derivatization using 1,2-diaminobenzene MS (ESI): Calcd for $[C_{23}H_{32}N_2O_4 + H]^+$ 401.2440, found 401.2479.

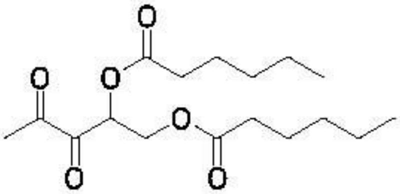

6-methyl-3,4-dioxoheptane-1,2-diyl diacetate (23): ^{1}H NMR ($CDCl_3$, 400 MHz) δ: 5.89–5.83 (m, 1H), 4.70–4.61 (m, 1H), 4.45–4.36 (m, 1H), 2.78–2.58 (m, 2H), 2.23–2.19 (m, 1H), 2.19 (s, 3H), 2.06 (s, 3H), 0.98 (d, J = 2.7 Hz, 3H), 0.97 (d, J = 2.7 Hz, 3H). ^{13}C NMR ($CDCl_3$, 100 MHz) δ: 198.2, 191.0, 171.2, 170.4, 73.2, 62.7, 45.0, 24.4, 22.9, 21.0, 20.7. Derivatization using 1,2-diaminobenzene MS (ESI): Calcd for $[C_{18}H_{22}N_2O_4 + H]^+$ 331.1658, found 331.1740.

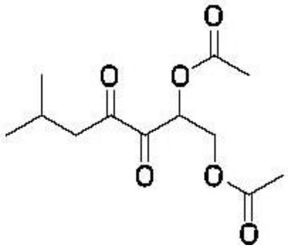

6-methyl-3,4-dioxoheptane-1,2-diyl dibutyrate (24): ^{1}H NMR ($CDCl_3$, 400 MHz) δ: 5.89–5.82 (m, 1H), 4.70–4.62 (m, 1H), 4.46–4.37 (m, 1H), 2.77–2.58 (m, 2H), 2.46–2.34 (m, 2H), 2.33–2.24 (m, 2H), 2.23–2.12 (m, 1H), 1.74–1.58 (m, 4H), 1.02–0.90 (m, 12H). ^{13}C NMR ($CDCl_3$, 100 MHz) δ: 198.3, 191.1, 173.8, 173.1, 62.5, 45.0, 36.1, 35.9, 24.4, 22.9, 22.8, 18.7, 18.6, 13.9. Derivatization using 1,2-diaminobenzene MS (ESI): Calcd for $[C_{22}H_{30}N_2O_4 + H]^+$ 387.2284, found 387.2244.

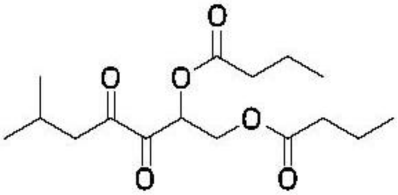

6-methyl-3,4-dioxoheptane-1,2-diyl dipentanoate (25): ^{1}H NMR ($CDCl_3$, 400 MHz) 5.89–5.82 (m, 1H), 4.70–4.61 (m, 1H), 4.46–4.37 (m, 1H), 2.78–2.58 (m, 2H), 2.48–2.38 (m, 2H), 2.35–2.25 (m, 2H), 2.25–2.13 (m, 1H), 1.68–1.54 (m, 4H), 1.43–1.30 (m, 4H), 1.01–0.89 (m, 12H). δ. 13C NMR (CDCl3, 100 MHz) δ: 198.3, 191.1, 174.0, 173.3, 62.5, 45.0, 34.0, 27.2, 22.9, 22.5, 14.1, 14.0. Derivatization using 1,2-diaminobenzene MS (ESI): Calcd for $[C_{24}H_{34}N_2O_4 + H]^+$ 415.2597, found 415.2499.

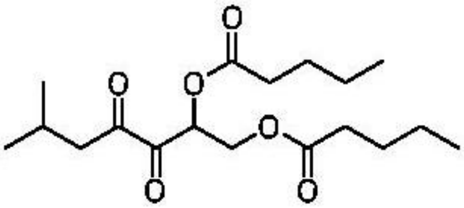

6-methyl-3,4-dioxoheptane-1,2-diyl dihexanoate (26): 1H NMR (CDCl3, 400 MHz) δ: 5.89–5.82 (m, 1H), 4.70–4.60 (m, 1H), 4.46–4.36 (m, 1H), 2.77–2.57 (m, 2H), 2.33–2.25 (m, 2H), 2.23–2.12 (m, 1H), 1.70–1.54 (m, 4H), 1.39–1.25 (m, 8H), 1.02–0.95 (m, 6H), 0.95–0.87 (m, 6H). 13C NMR (CDCl3, 100 MHz) δ: 198.3, 191.1, 174.0, 173.3, 62.5, 34.2, 31.5, 24.8, 22.9, 22.7, 14.3. Derivatization using 1,2-diaminobenzene MS (ESI): Calcd for $[C_{26}H_{38}N_2O_4 + H]^+$ 443.2910, found 443.2855.

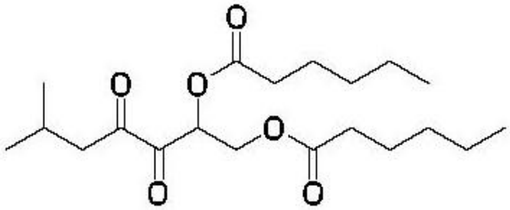

3,4-dioxodecane-1,2-diyl diacetate (27): ^{1}H NMR ($CDCl_3$, 400 MHz) δ: 5.90–5.84 (m, 1H), 4.71–4.64 (m, 1H), 4.45–4.36 (m, 1H), 2.87–2.72 (m, 2H), 2.19 (s, 3H), 2.17 (s, 3H), 1.68–1.58 (m, 2H), 1.38–1.27 (m, 6H), 0.94–0.87 (m, 3H). ^{13}C NMR ($CDCl_3$, 100 MHz) δ: 198.7, 190.9, 171.2, 170.4, 73.2, 62.7, 36.5, 31.9, 29.1, 23.1, 22.9, 21.0, 20.8, 14.4. Derivatization using 1,2-diaminobenzene MS (ESI): Calcd for $[C_{20}H_{26}N_2O_4 + H]^+$ 359.1971, found 359.1903.

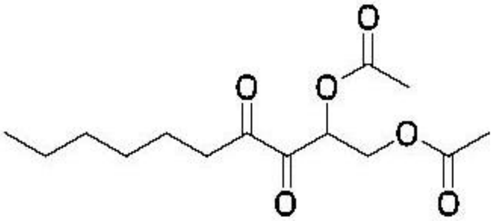

3,4-dioxodecane-1,2-diyl dibutyrate (28): ^{1}H NMR ($CDCl_3$, 400 MHz) δ: 5.91–5.84 (m, 1H), 4.72–4.64 (m, 1H), 4.46–4.37 (m, 1H), 2.86–2.2.72 (m, 2H), 2.47–2.38 (m, 2H), 2.33–2.24 (m, 2H), 1.76–1.1.60 (m, 6H), 1.38–1.27 (m, 6H), 1.04–0.88 (m, 9H). ^{13}C NMR ($CDCl_3$, 100 MHz) δ: 198.7, 191.0, 173.9, 173.1, 73.1, 62.5, 36.5, 36.2, 35.9, 31.9, 29.1, 23.1, 22.9, 18.7, 14.4, 14.0. Derivatization using 1,2-diaminobenzene MS (ESI): Calcd for $[C_{24}H_{34}N_2O_4 + H]^+$ 415.2597, found 415.2528.

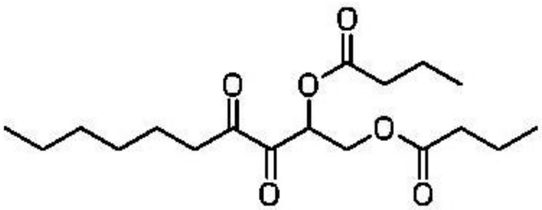

3,4-dioxodecane-1,2-diyl dipentanoate (29): ^{1}H NMR ($CDCl_3$, 400 MHz) δ: 5.90–5.83 (m, 1H), 4.71–4.63 (m, 1H), 4.45–4.37 (m, 1H), 2.85–2.72 (m, 2H), 2.48–2.40 (m, 2H), 2.40–2.34 (m, 2H), 2.34–2.27 (m, 2H), 1.69–1.57 (m, 6H), 1.45–1.30 (m, 8H), 0.97–0.89 (m, 9H). ^{13}C NMR ($CDCl_3$, 100 MHz) δ: 198.7, 191.0, 174.1, 173.3, 73.1, 62.5, 36.5, 34.0, 33.8, 31.9, 29.1, 27.2, 23.1, 22.9, 22.6, 22.5, 14.4, 14.1. Derivatization using 1,2-diaminobenzene MS (ESI): Calcd for $[C_{26}H_{38}N_2O_4 + H]^+$ 443.2910, found 443.2867.

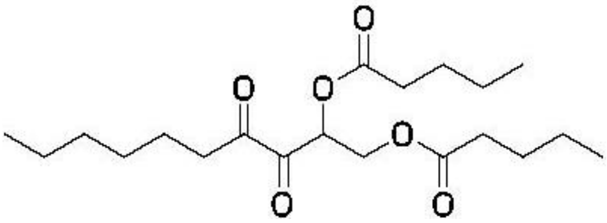

3,4-dioxodecane-1,2-diyl dihexanoate (30): ^{1}H NMR ($CDCl_3$, 400 MHz) δ: 5.90–5.83 (m, 1H), 4.72–4.62 (m, 1H), 4.45–4.36 (m, 1H), 2.86–2.71 (m, 2H), 2.48–2.40 (m, 2H), 2.40–2.33 (m, 2H), 2.33–2.25 (m, 2H), 1.71–1.58 (m, 6H), 1.39–1.27 (m, 12H), 0.96–0.87 (m, 9H). ^{13}C NMR ($CDCl_3$, 100 MHz) δ: 198.7, 191.0, 174.1, 173.3, 73.1, 62.5, 36.5, 34.3, 34.0, 31.9, 31.6, 31.5, 29.1, 24.8, 23.1, 22.9, 22.7, 14.4, 14.3. Derivatization using 1,2-diaminobenzene MS (ESI): Calcd for $[C_{28}H_{42}N_2O_4 + H]^+$ 471.3223, found 471.3176.

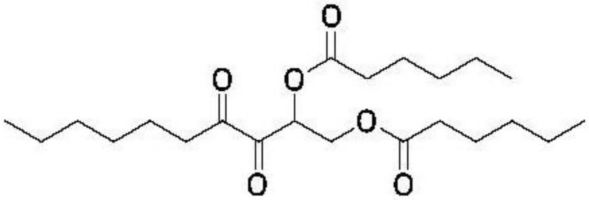

Figure S1. ^{1}H NMR (top) and ^{13}C NMR (bottom) spectra of 3-diazo-4-oxopentane-1,2-diyl diacetate (7).

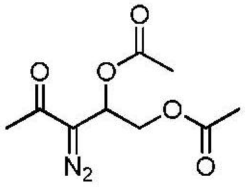

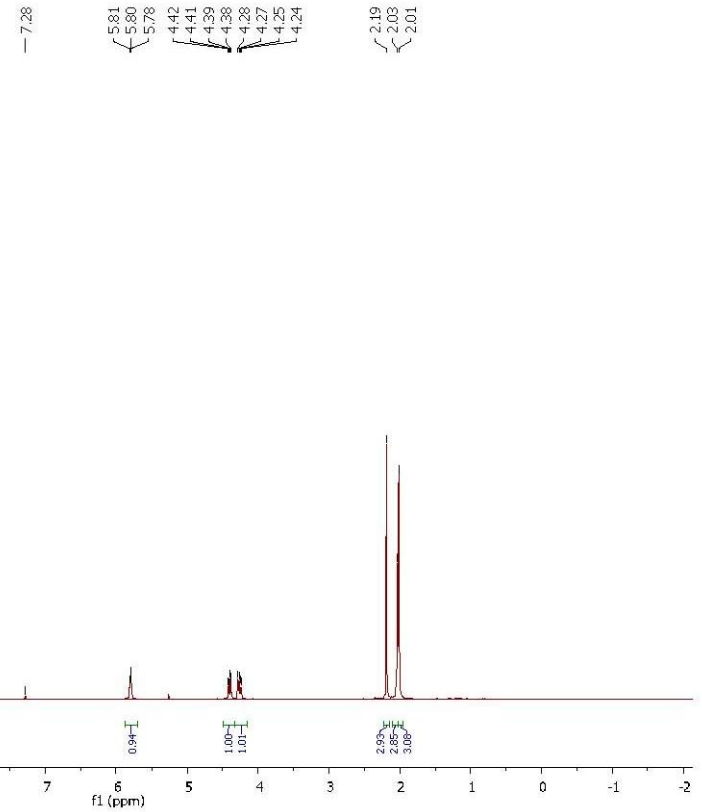

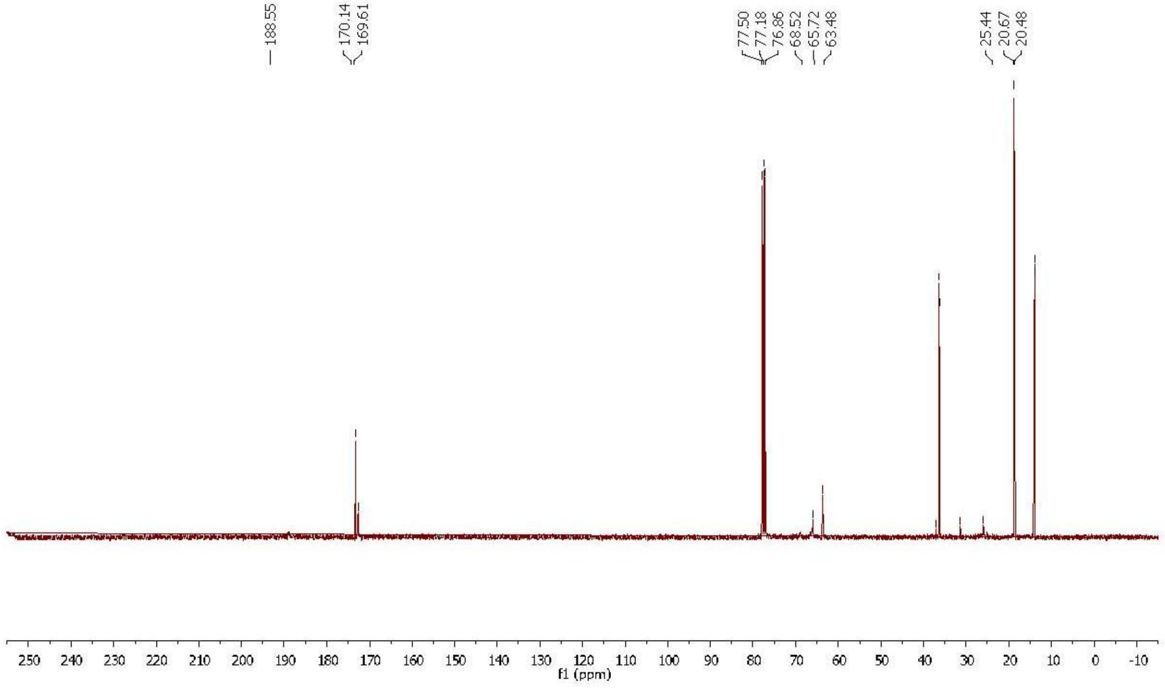

Figure S2. ^{1}H NMR (top) and ^{13}C NMR (bottom) spectra of 3-diazo-4-oxopentane-1,2-diyl dibutyrate (8).

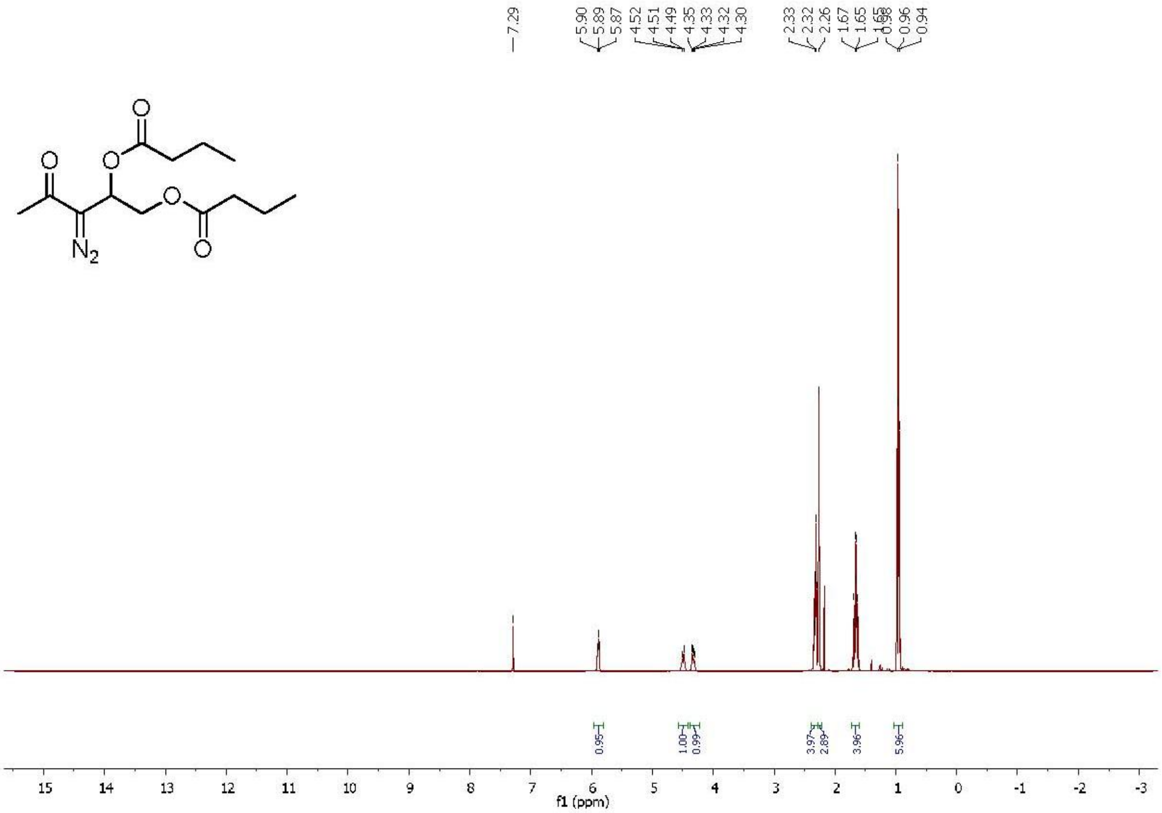

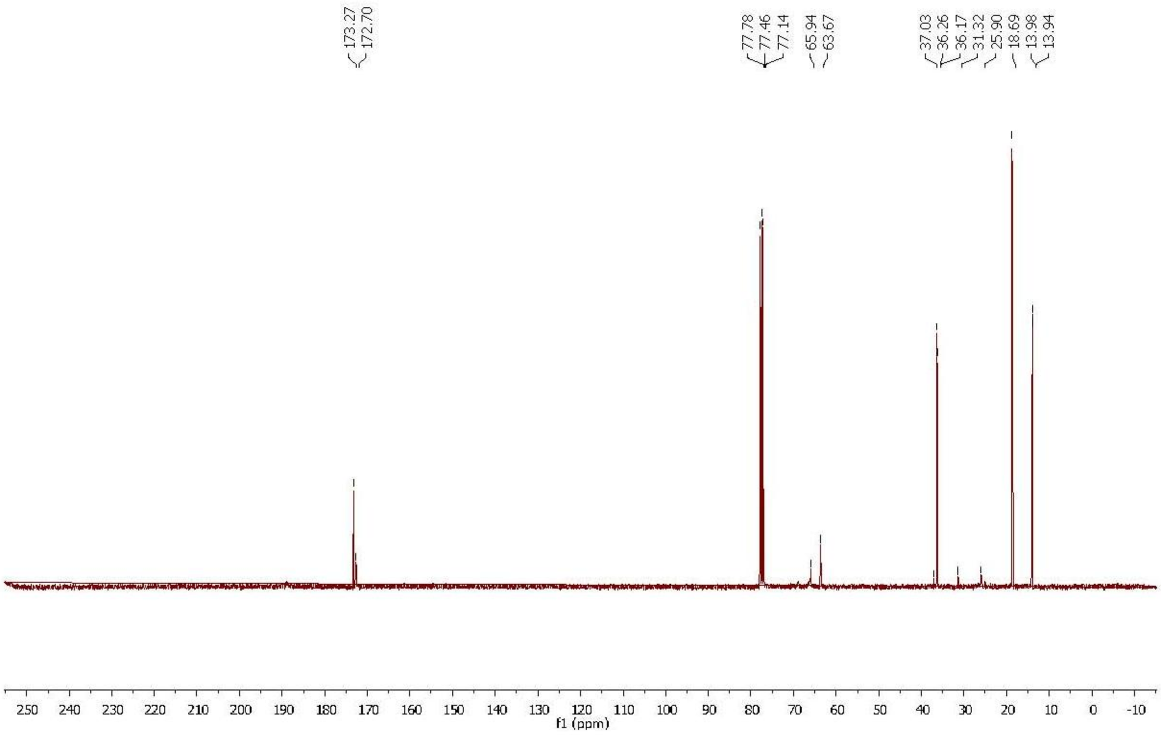

Figure S3. ^{1}H NMR (top) and ^{13}C NMR (bottom) spectra of 3-diazo-4-oxopentane-1,2-diyl dipentanoate (9).

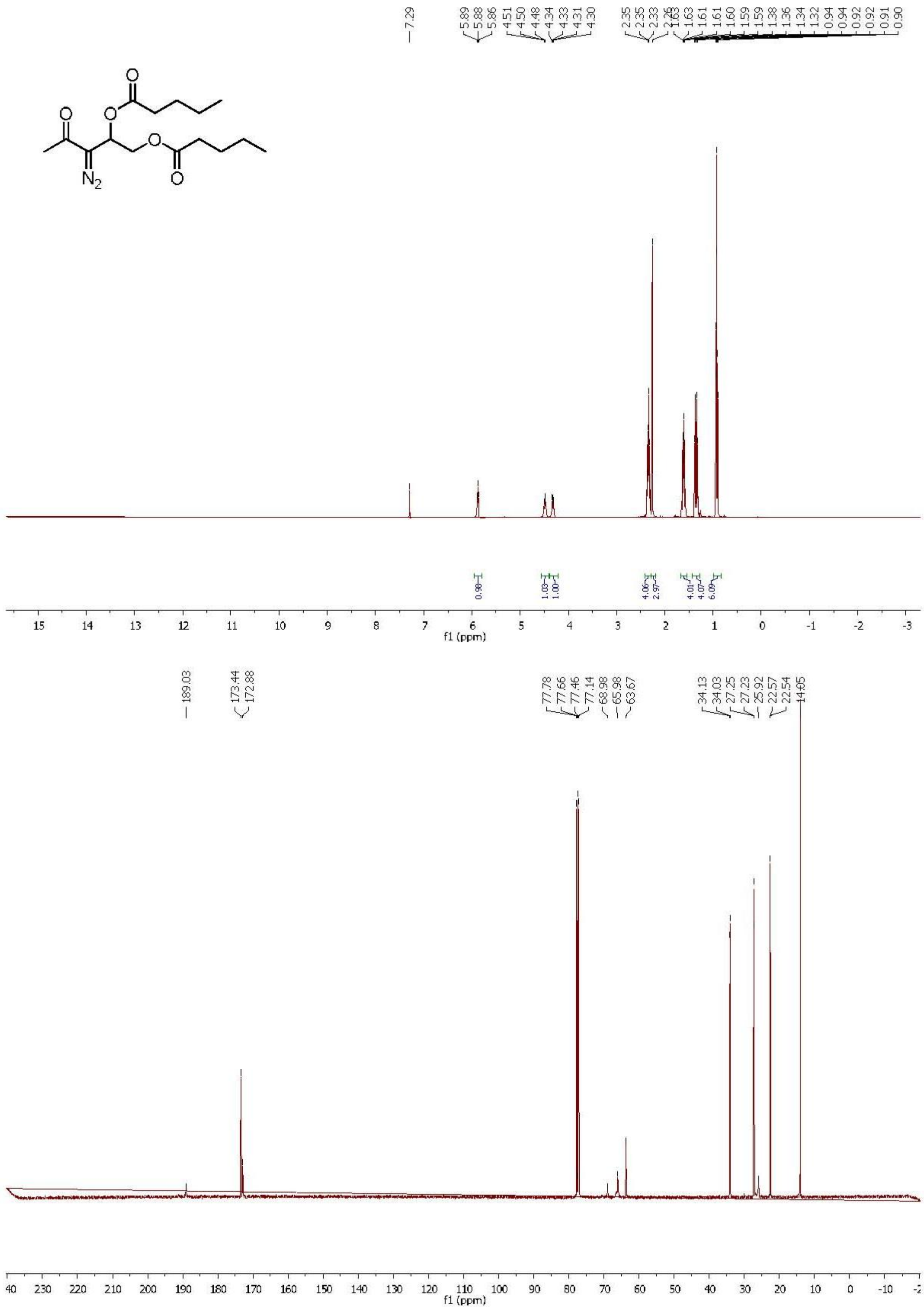

Figure S4. ^{1}H NMR (top) and ^{13}C NMR (bottom) spectra of 3-diazo-4-oxopentane-1,2-diyl dihexanoate (10).

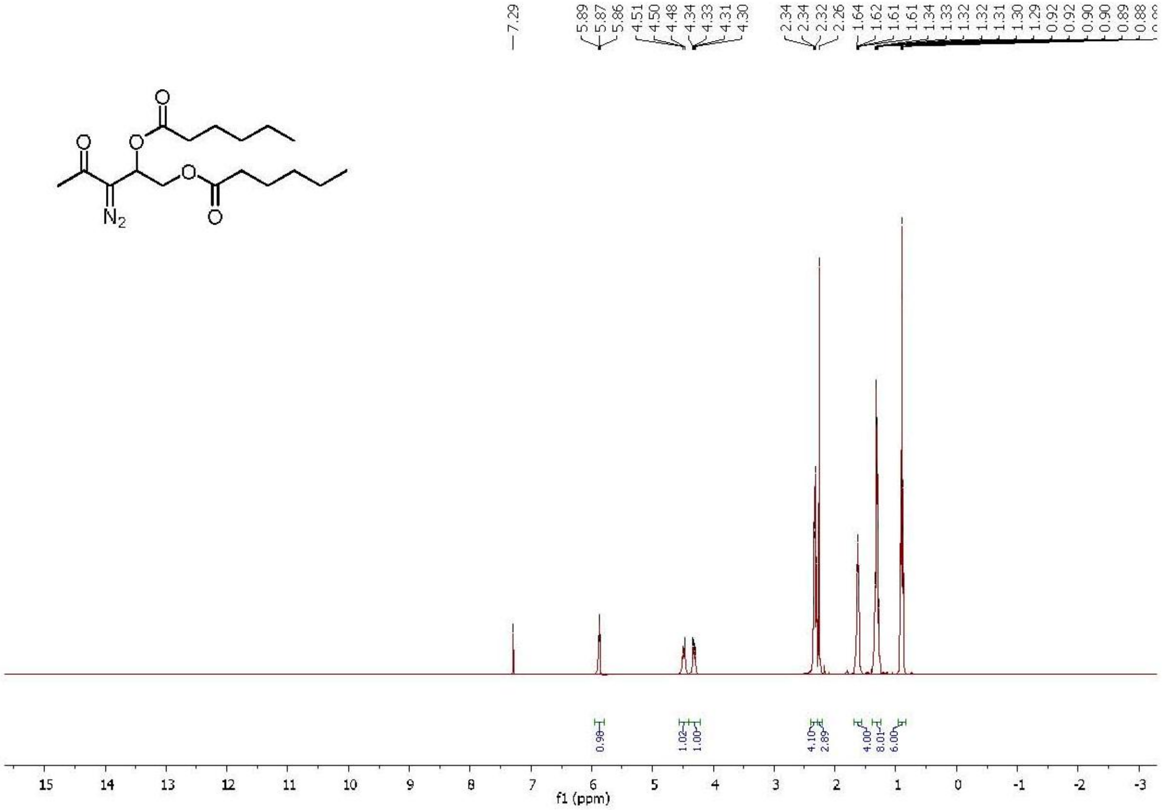

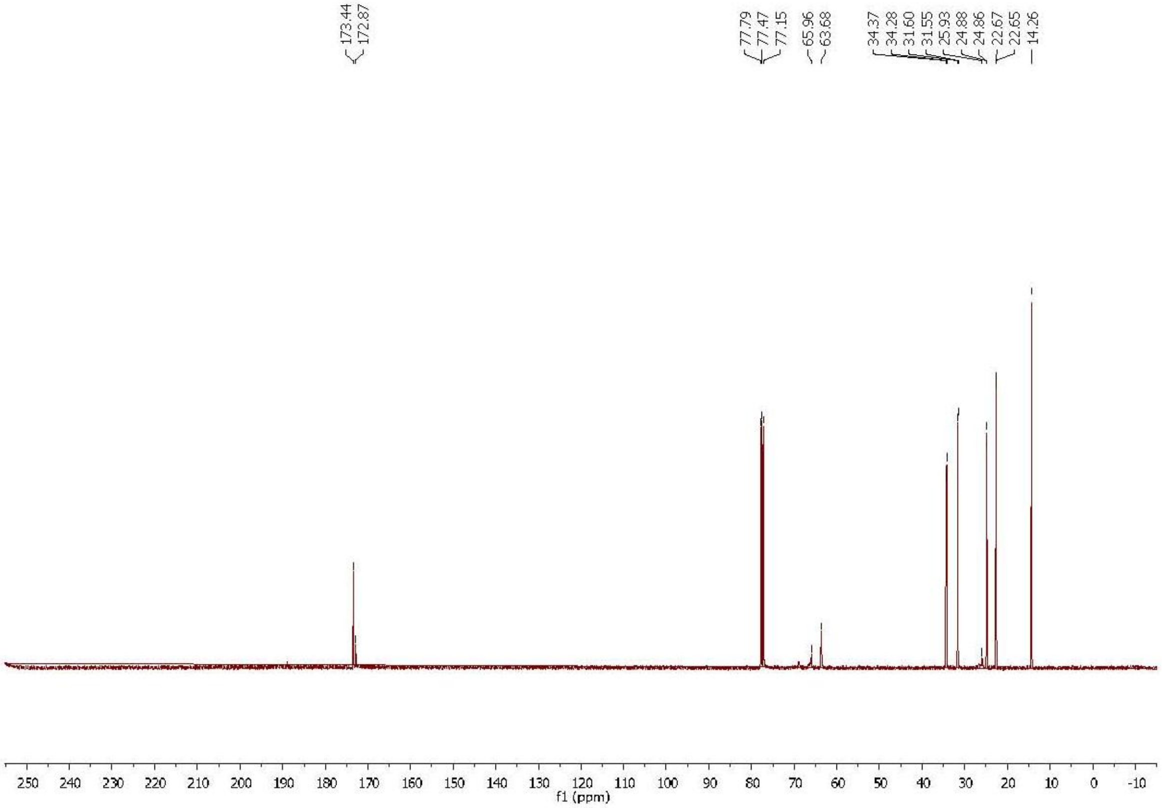

Figure S5. ^{1}H NMR (top) and ^{13}C NMR (bottom) spectra of 3-diazo-6-methyl-4-oxoheptane-1,2-diyl diacetate (11).

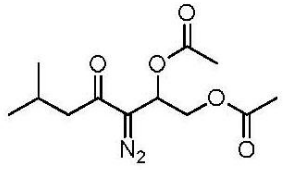

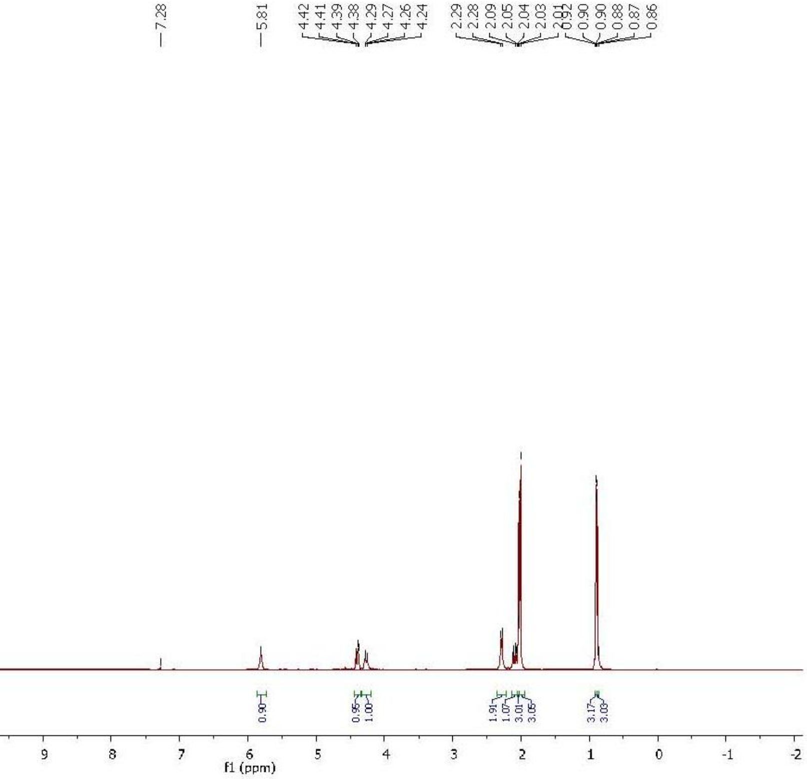

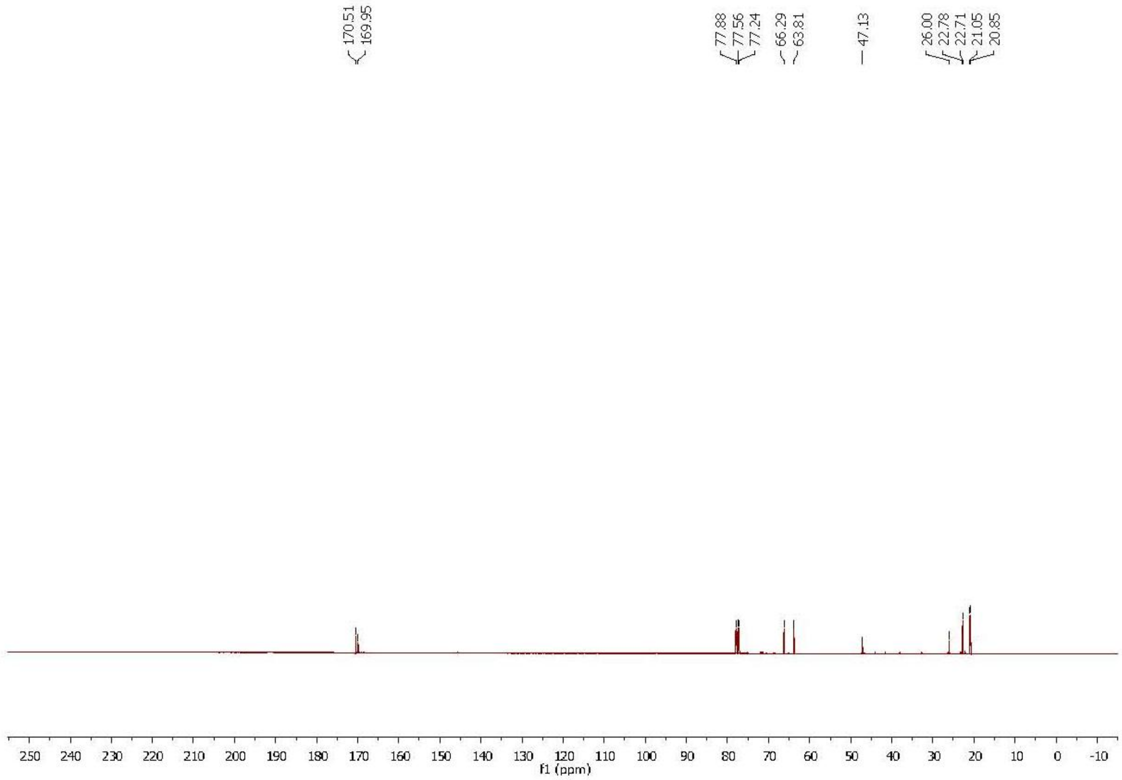

Figure S6. ^{1}H NMR (top) and ^{13}C NMR (bottom) spectra of 3-diazo-6-methyl-4-oxoheptane-1,2-diyl dibutyrate (12).

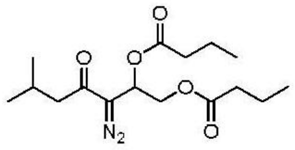

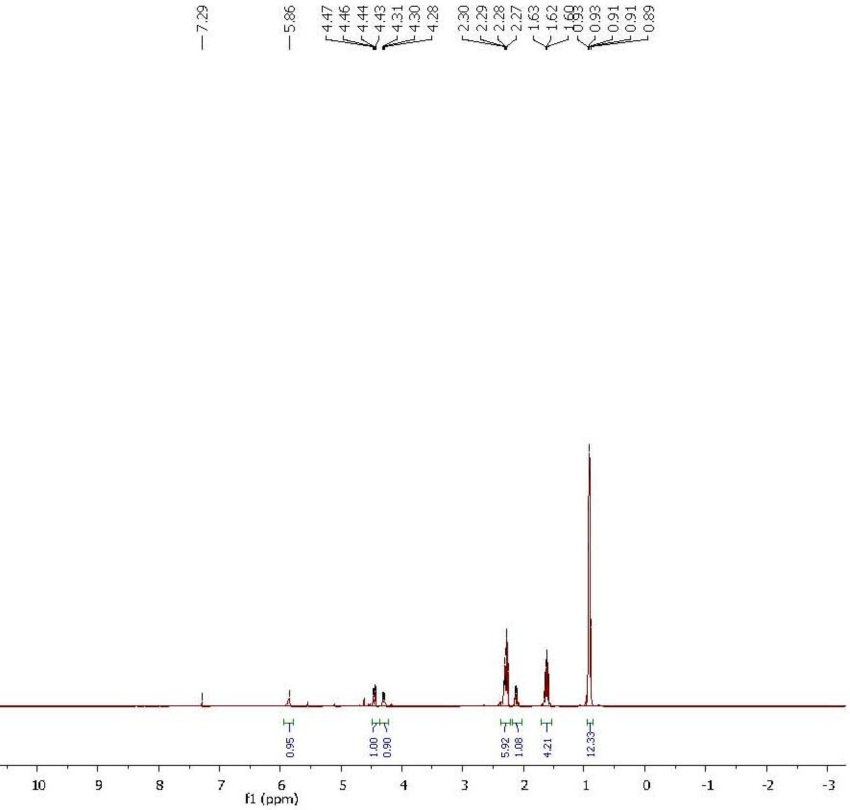

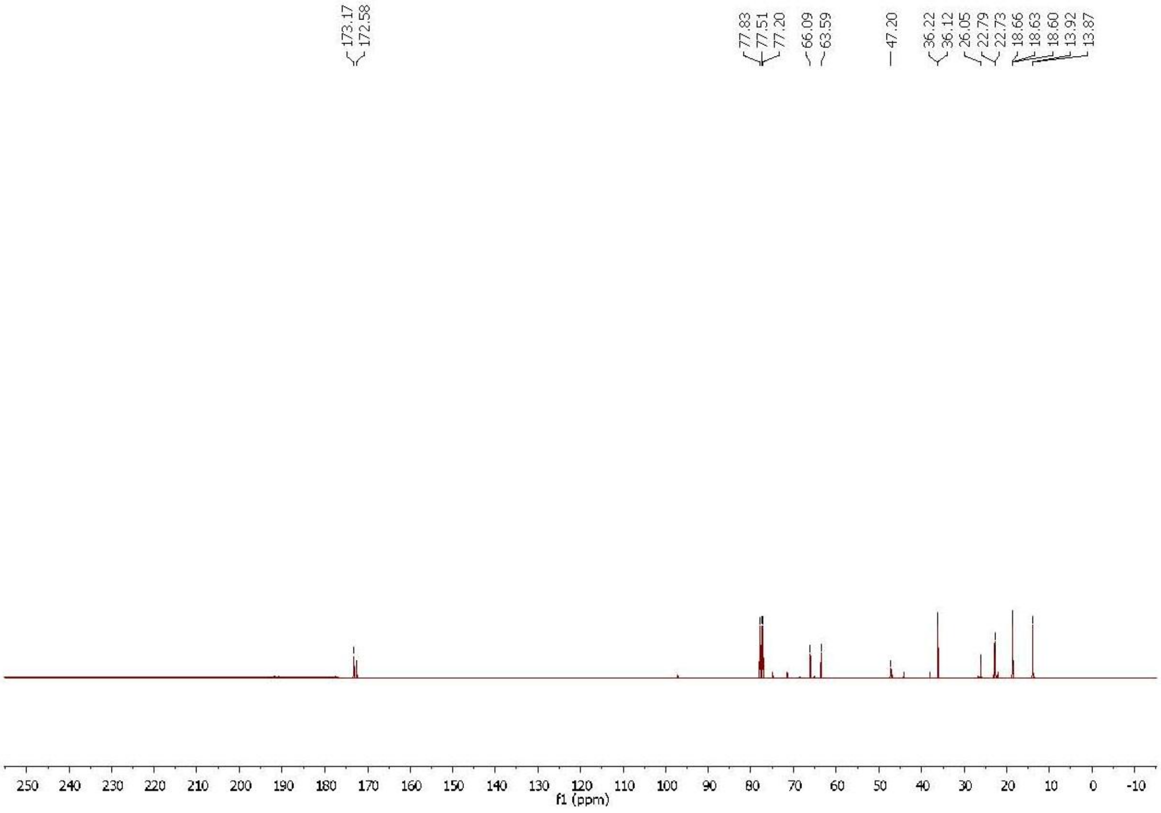

Figure S7. ^{1}H NMR (top) and ^{13}C NMR (bottom) spectra of 3-diazo-6-methyl-4-oxoheptane-1,2-diyl dipentanoate (13).

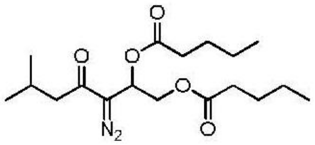

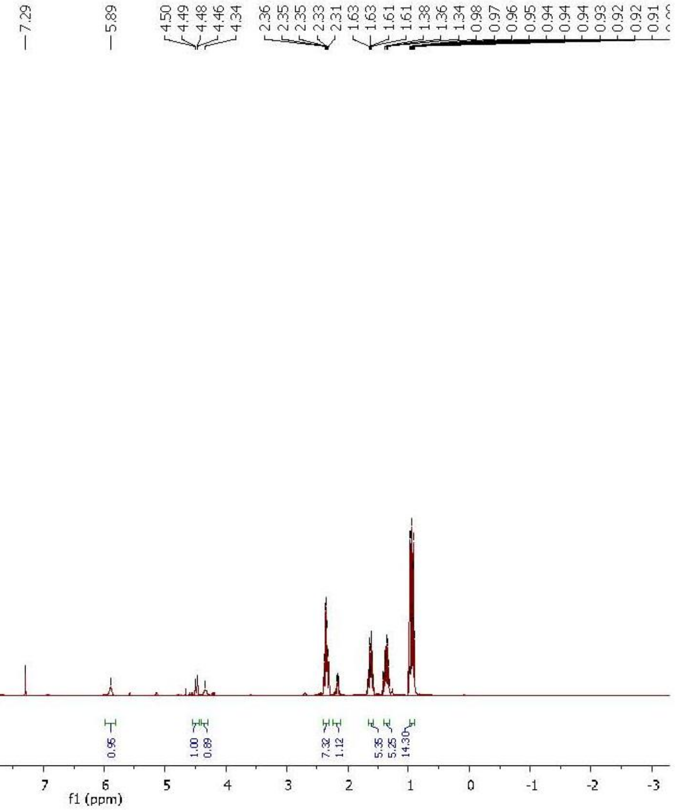

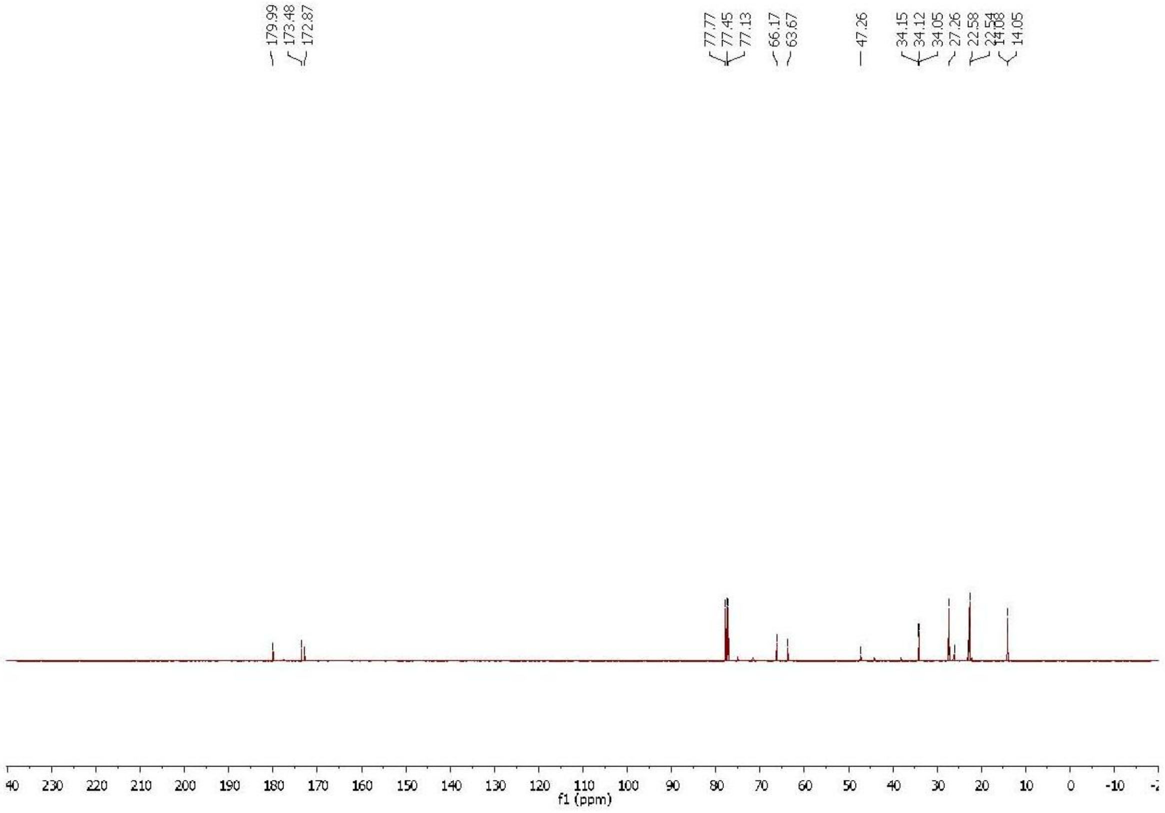

Figure S8. ^{1}H NMR (top) and ^{13}C NMR (bottom) spectra of 3-diazo-6-methyl-4-oxoheptane-1,2-diyl dihexanoate (14).

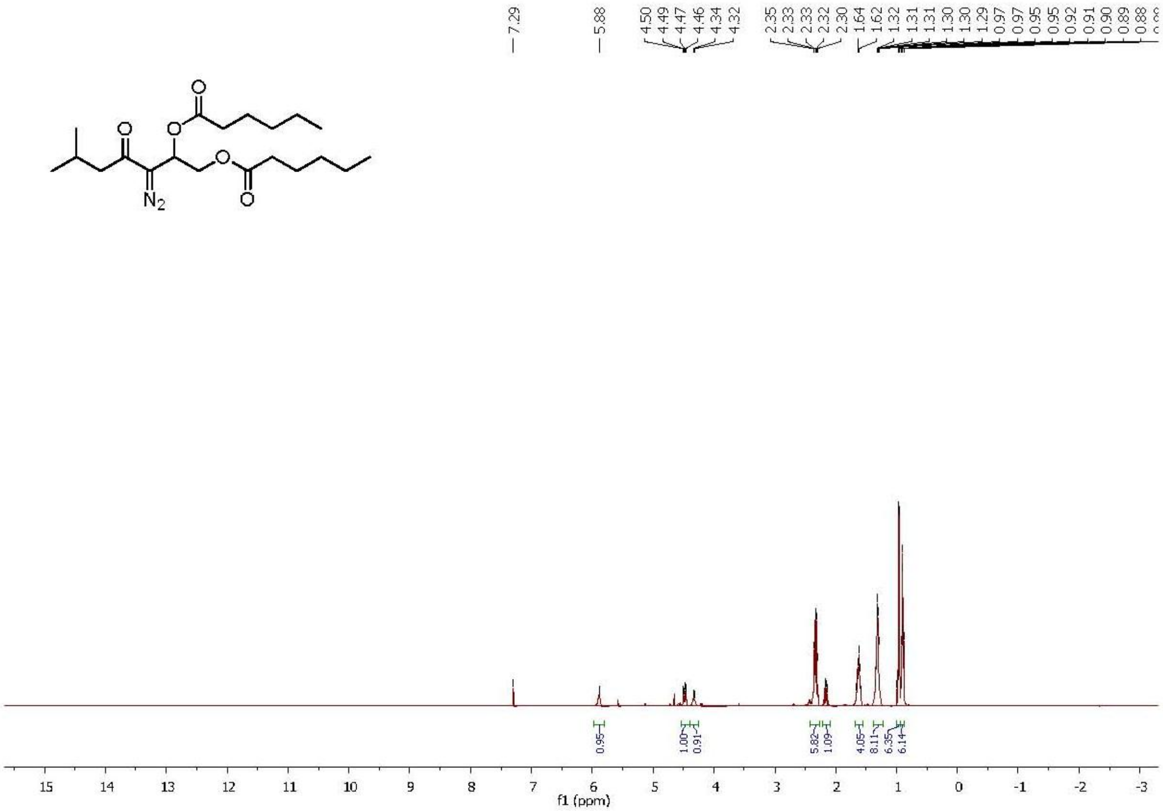

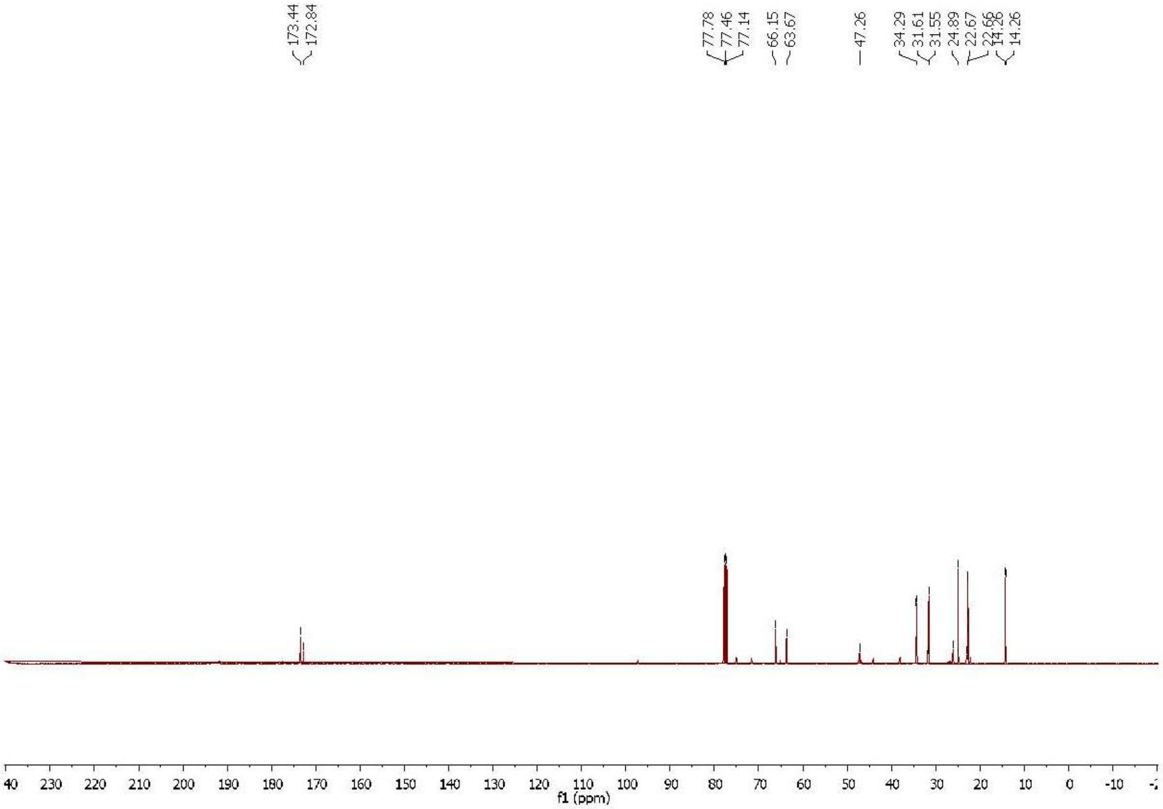

Figure S9. ^{1}H NMR (top) and ^{13}C NMR (bottom) spectra of 3-diazo-4-oxodecane-1,2-diyl diacetate (15).

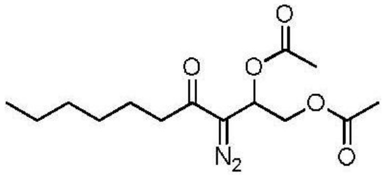

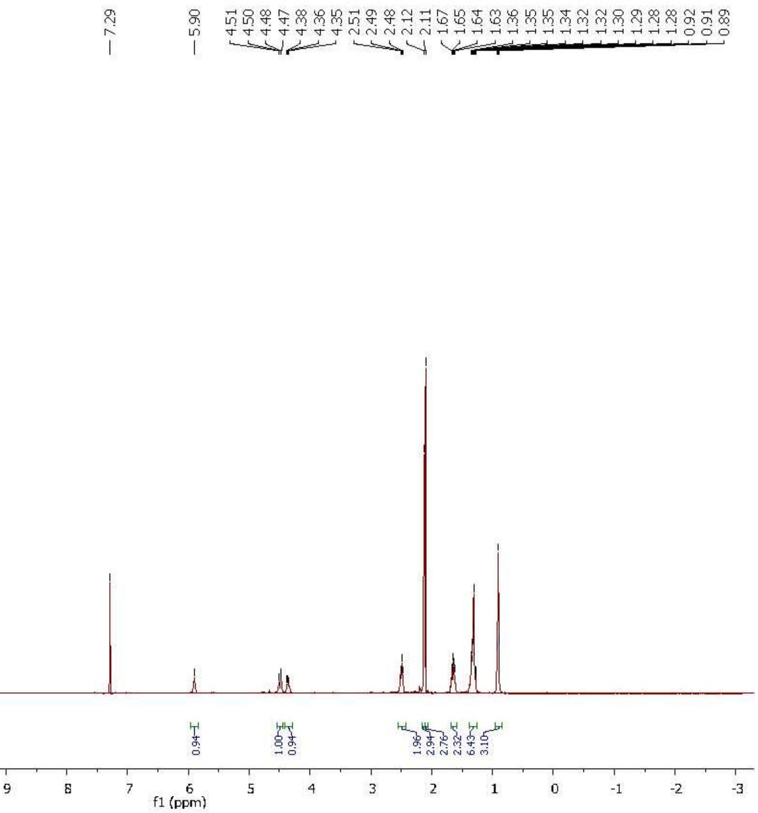

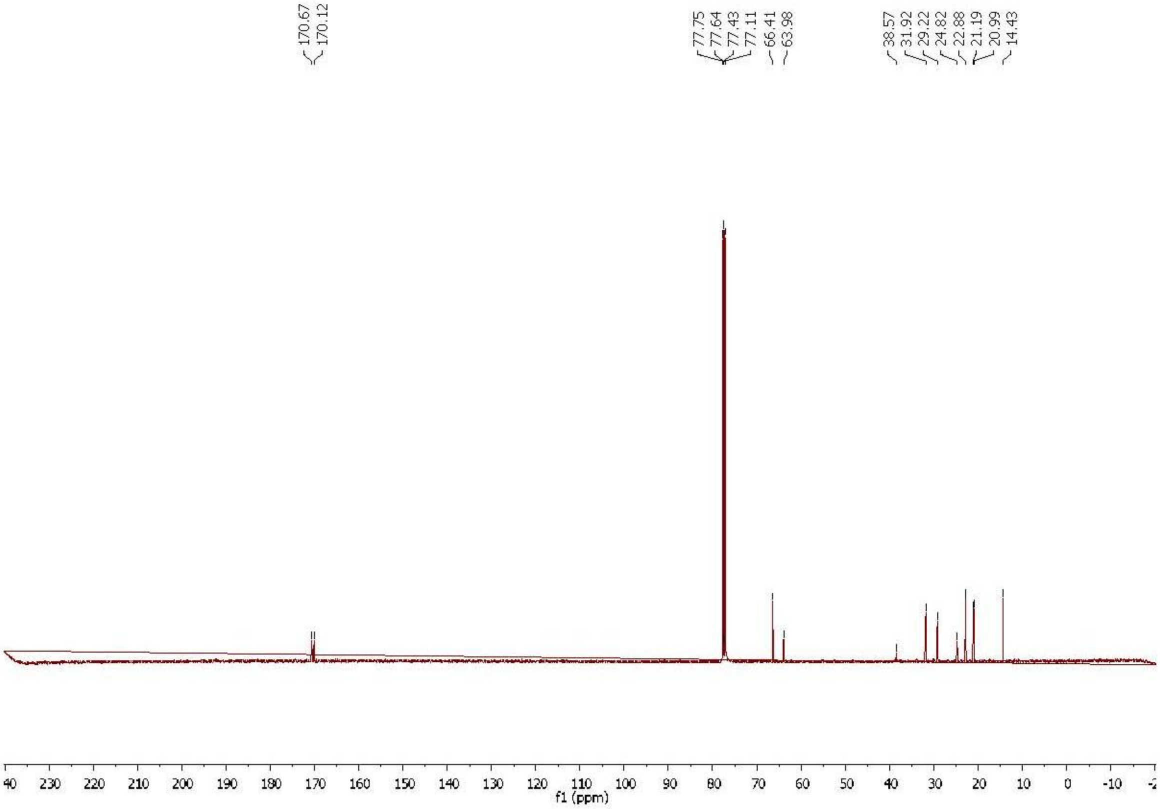

Figure S10. ^{1}H NMR (top) and ^{13}C NMR (bottom) spectra of 3-diazo-4-oxodecane-1,2-diyl dibutyrate (16).

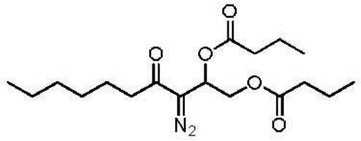

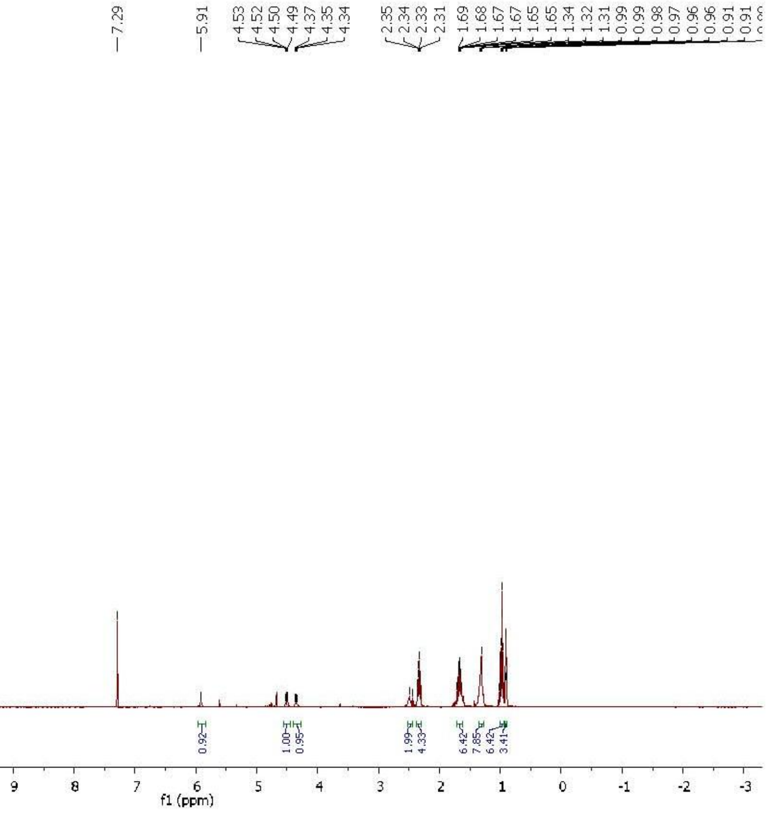

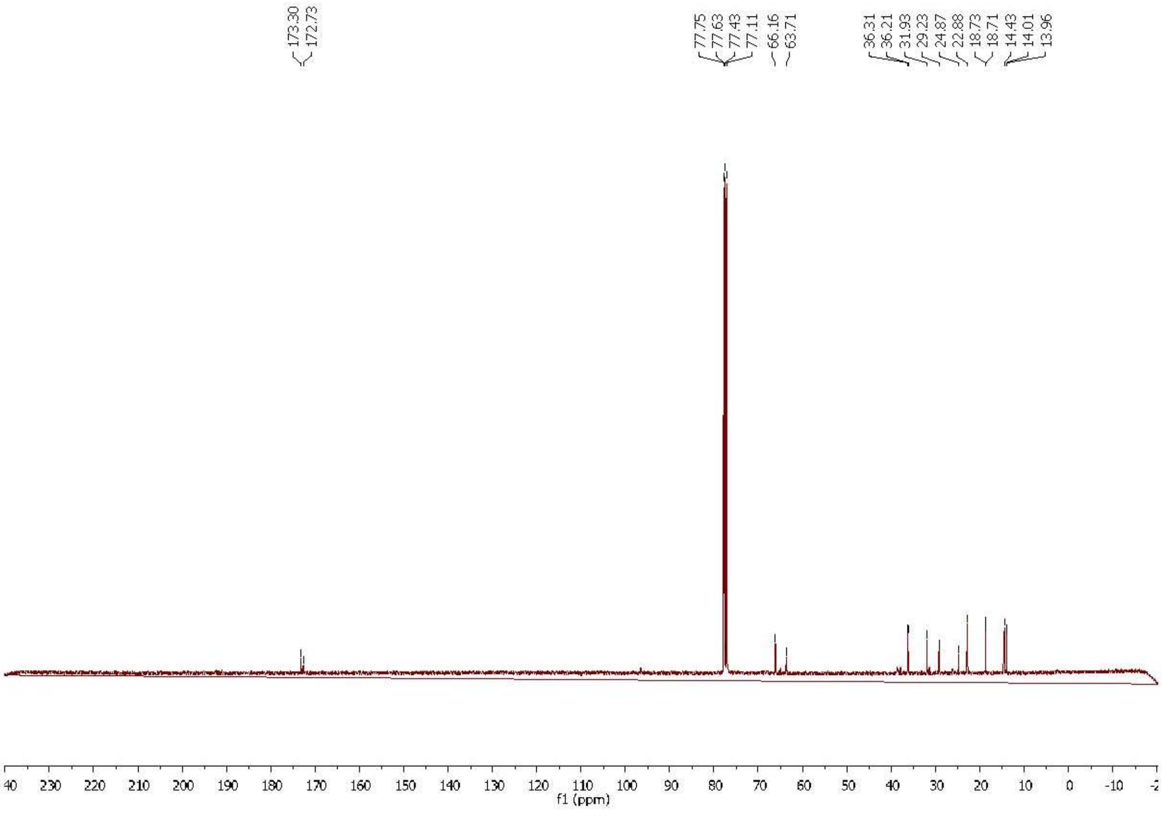

Figure S11. ^{1}H NMR (top) and ^{13}C NMR (bottom) spectra of 3-diazo-4-oxodecane-1,2-diyl dipentanoate (17).

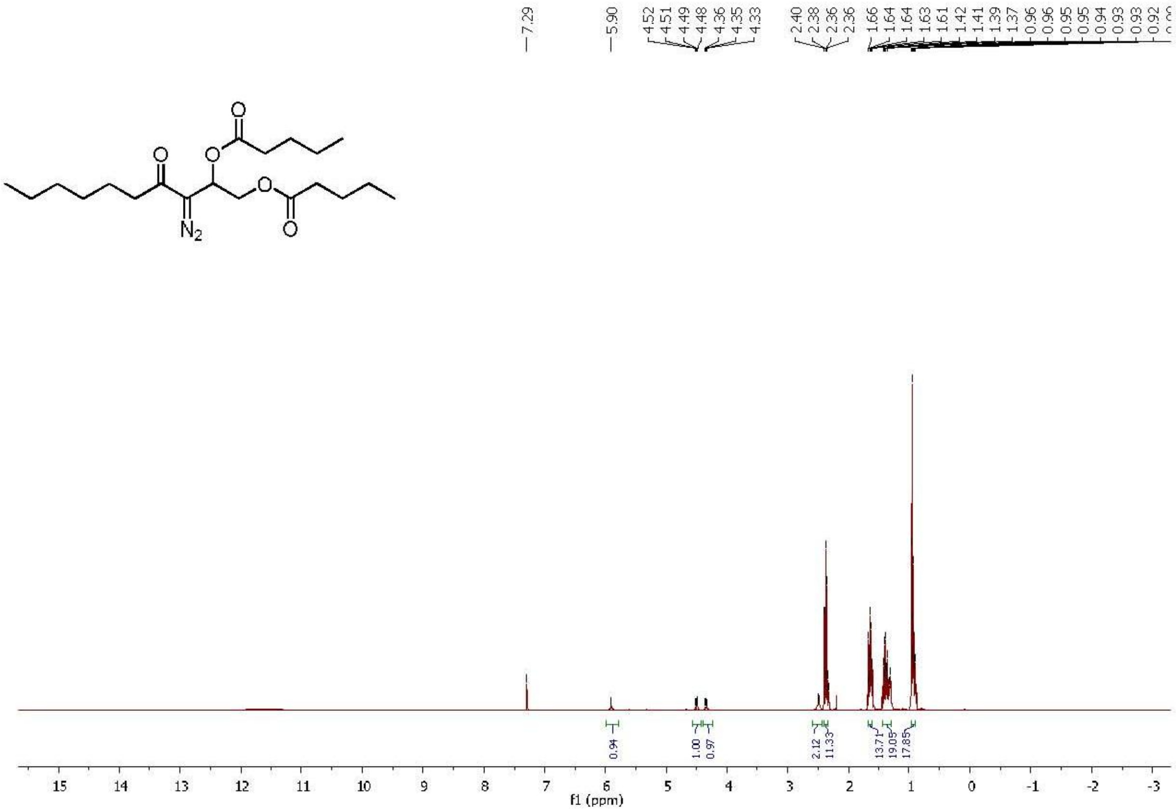

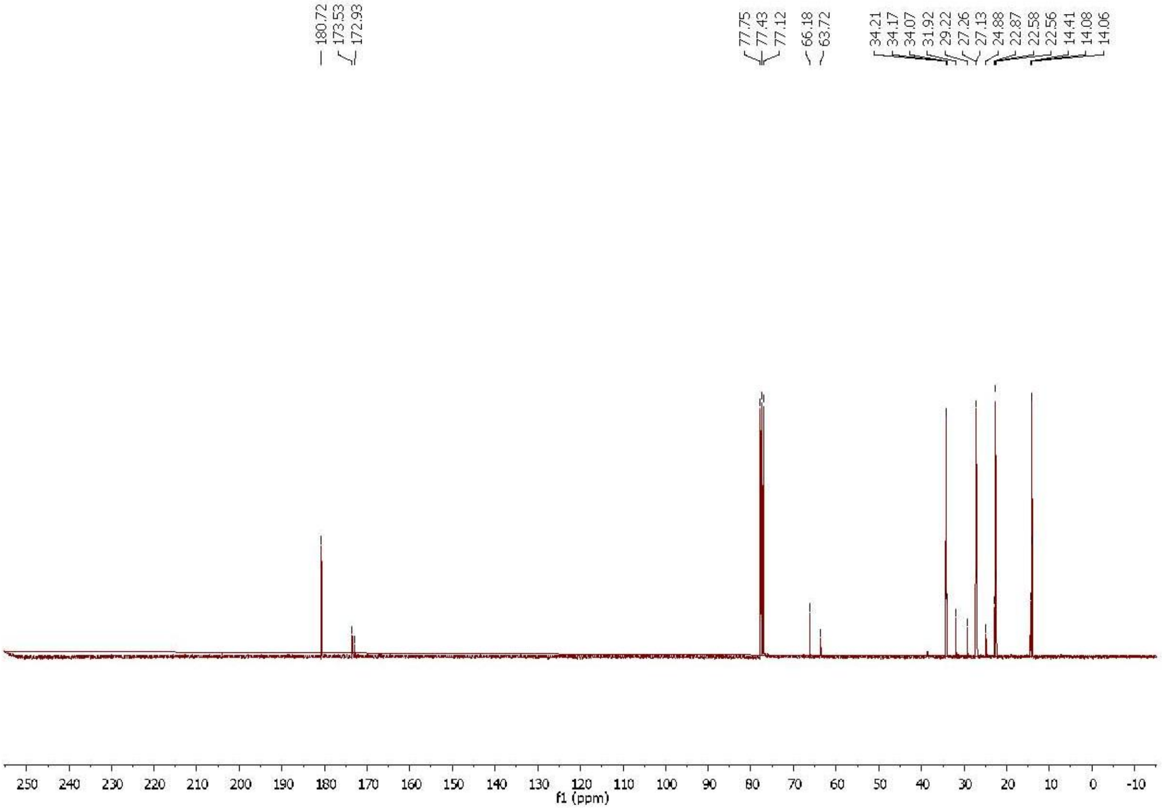

Figure S12. ^{1}H NMR (top) and ^{13}C NMR (bottom) spectra of 3-diazo-4-oxodecane-1,2-diyl dihexanoate (18).

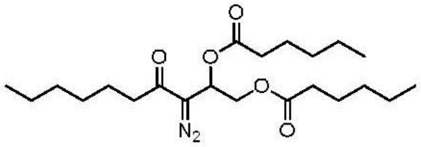

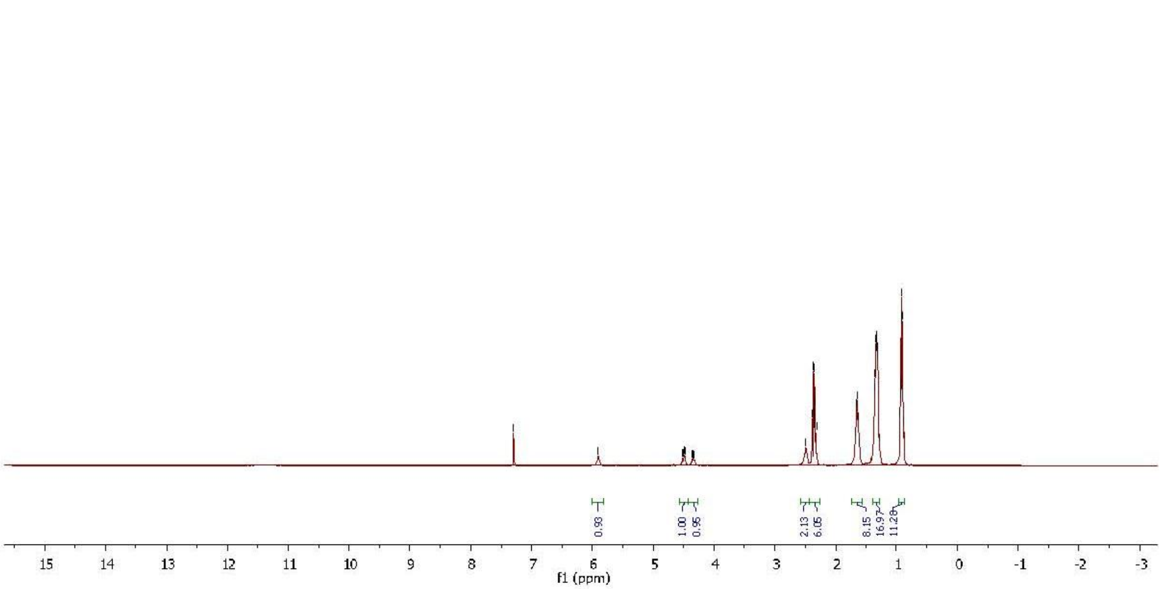

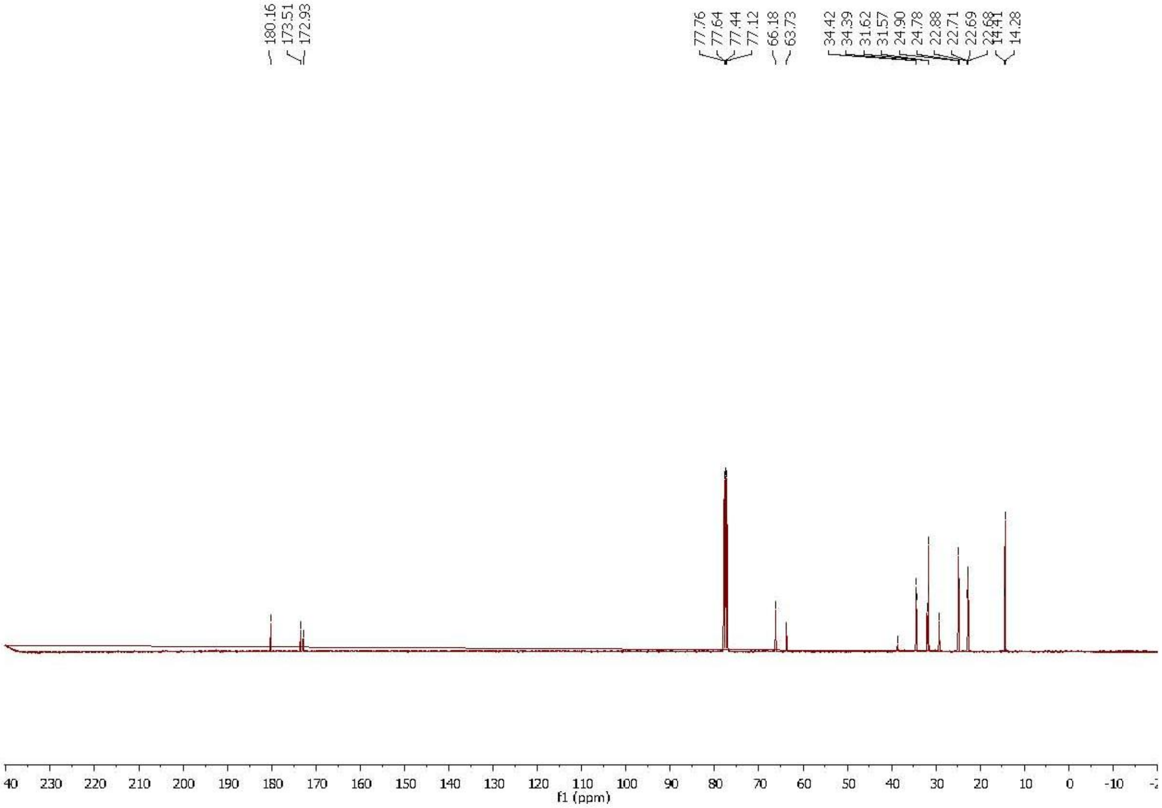

Figure S13. ^{1}H NMR (top) and ^{13}C NMR (bottom) spectra of 3,4-dioxopentane-1,2-diyl diacetate (19).

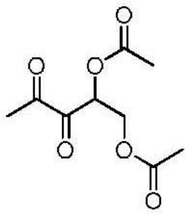

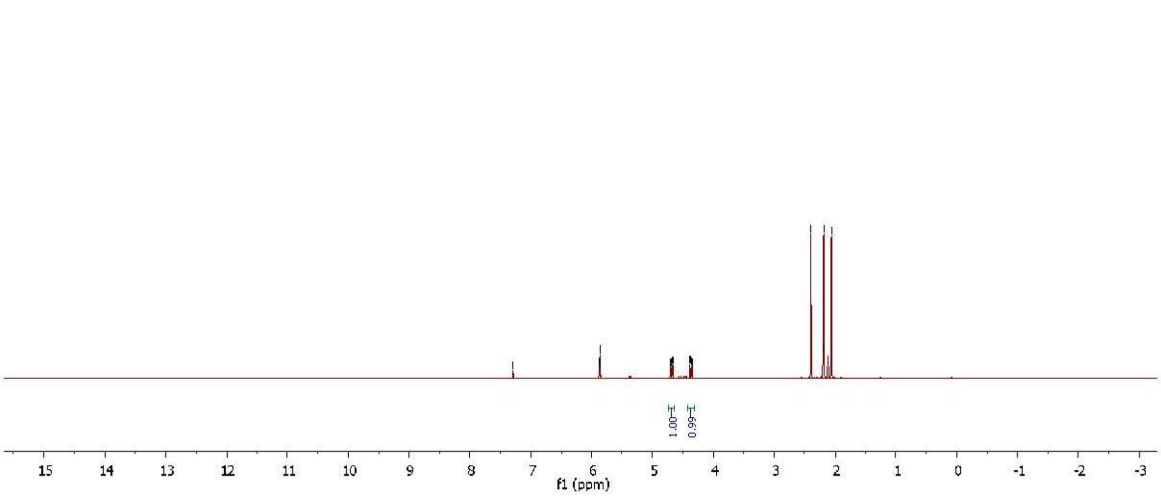

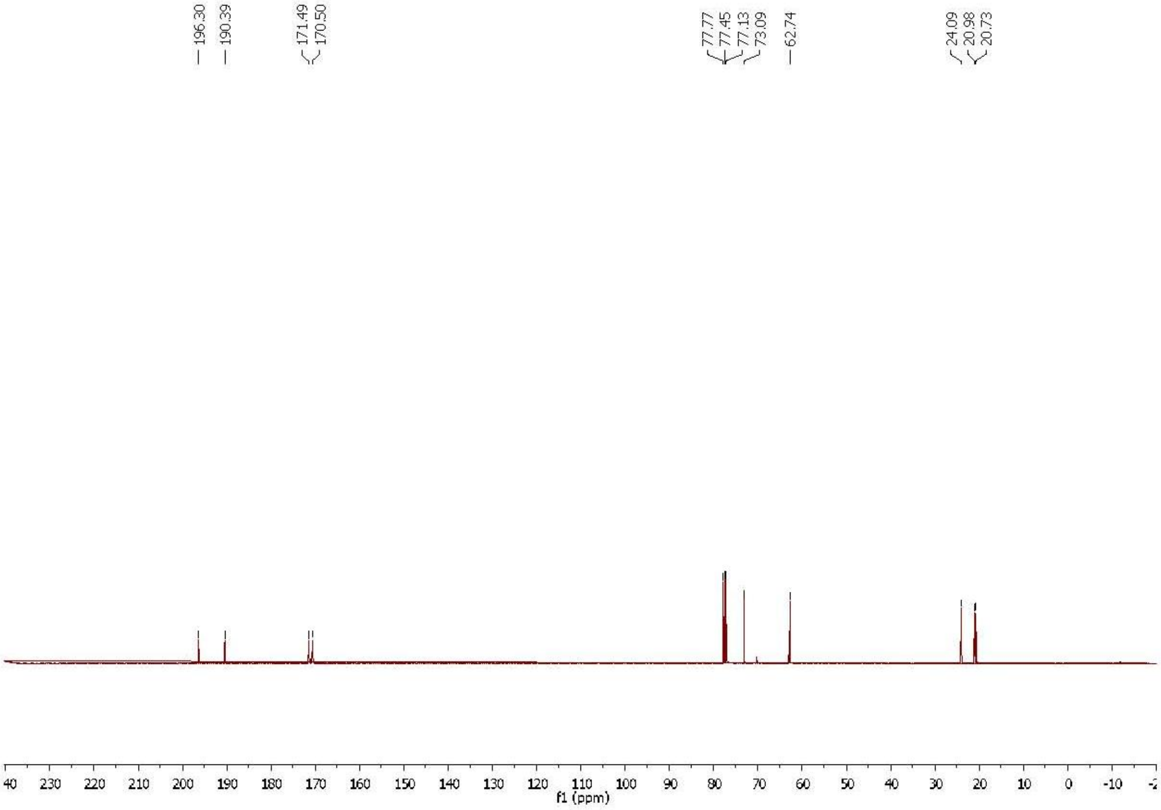

Figure S14. ^{1}H NMR (top) and ^{13}C NMR (bottom) spectra of 3,4-dioxopentane-1,2-diyl dibutyrate (20).

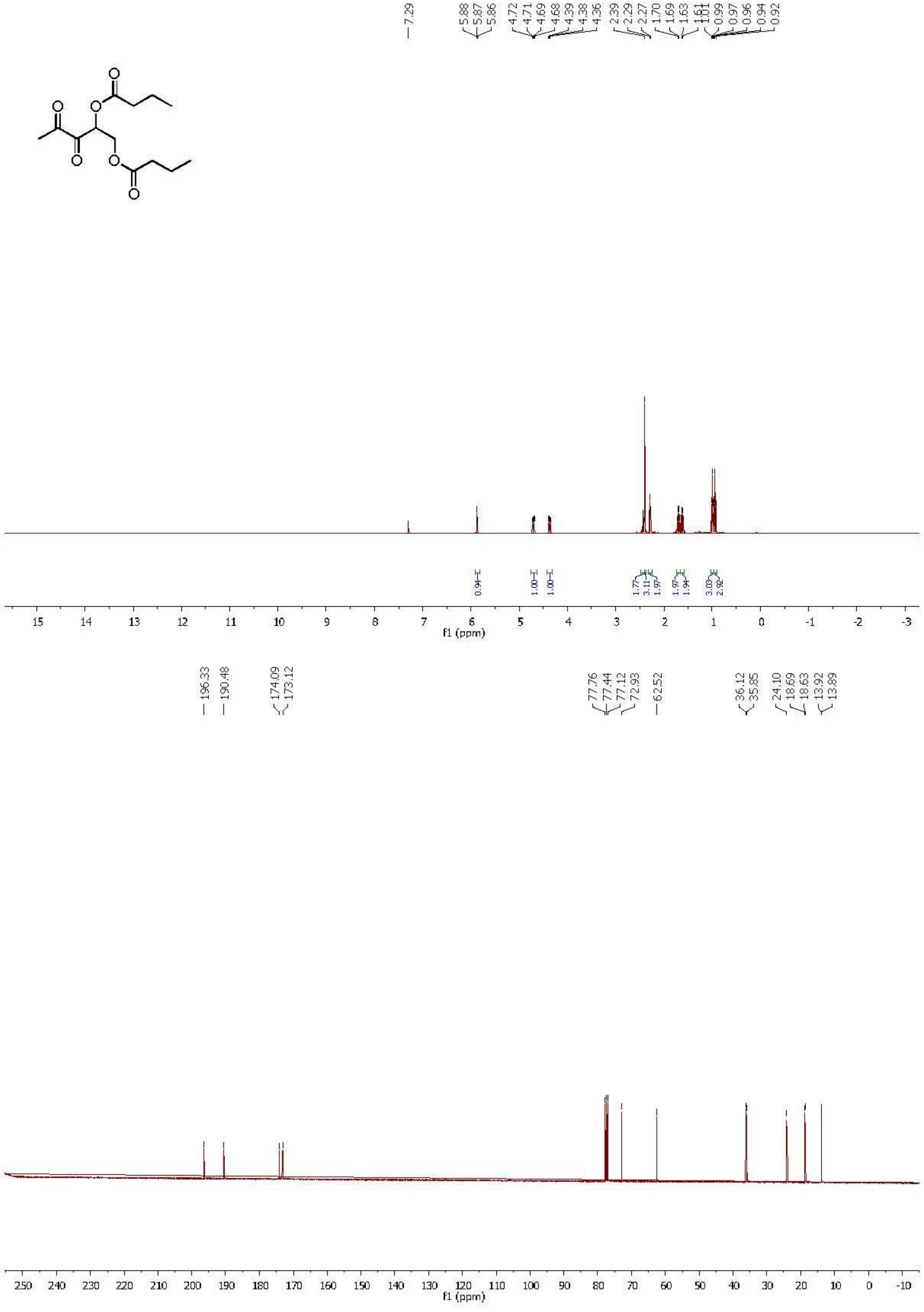

Figure S15. ^{1}H NMR (top) and ^{13}C NMR (bottom) spectra of 3,4-dioxopentane-1,2-diyl dipentanoate (21).

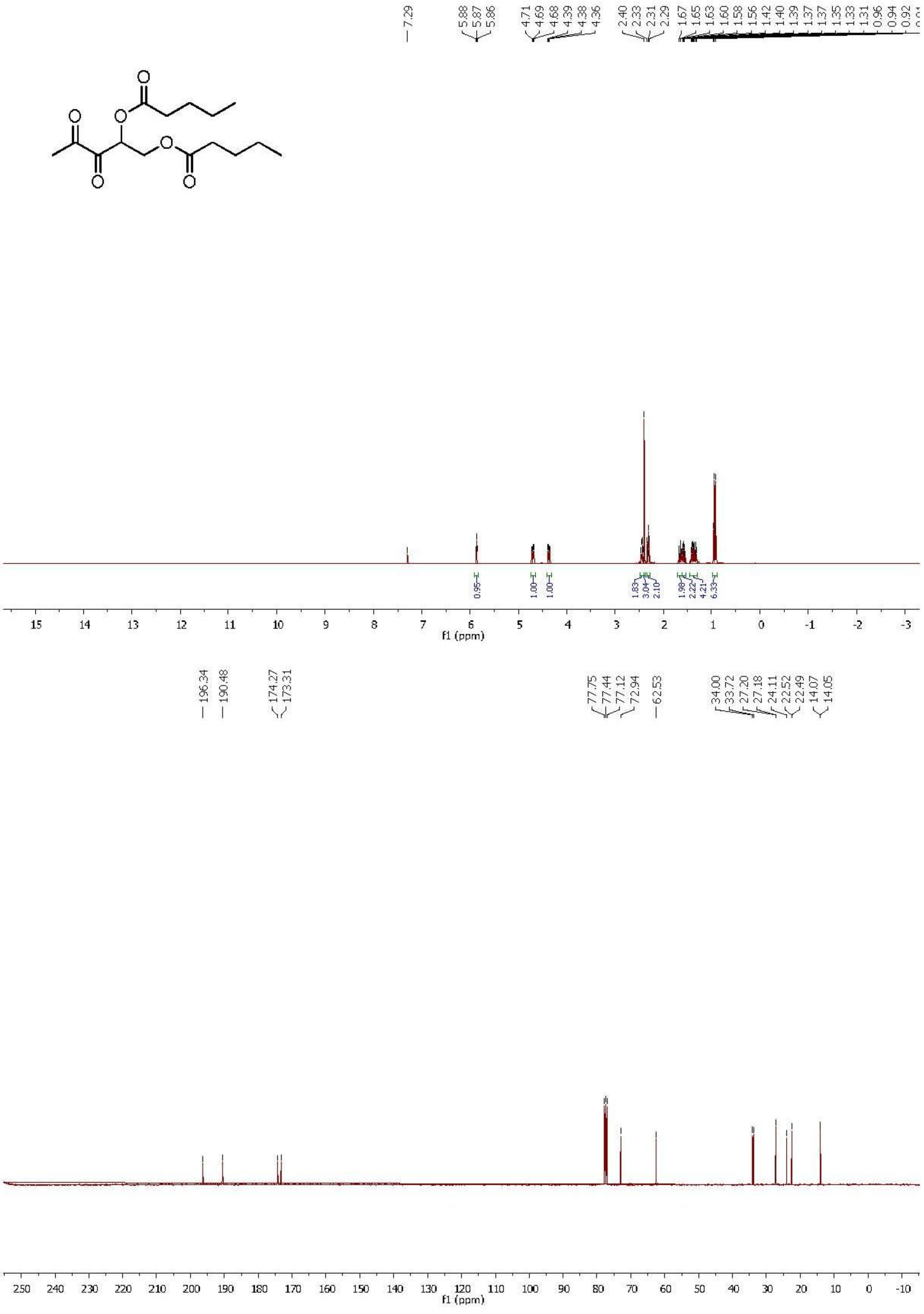

Figure S16. ^{1}H NMR (top) and ^{13}C NMR (bottom) spectra of 3,4-dioxopentane-1,2-diyl dihexanoate (22).

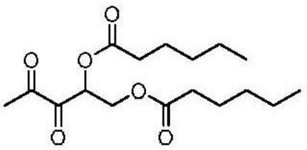

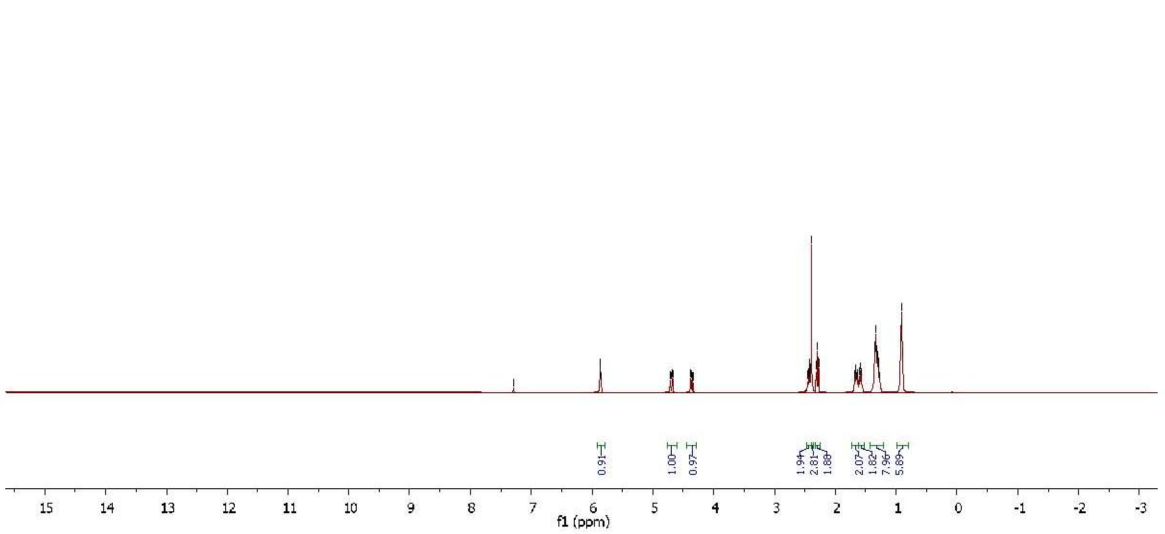

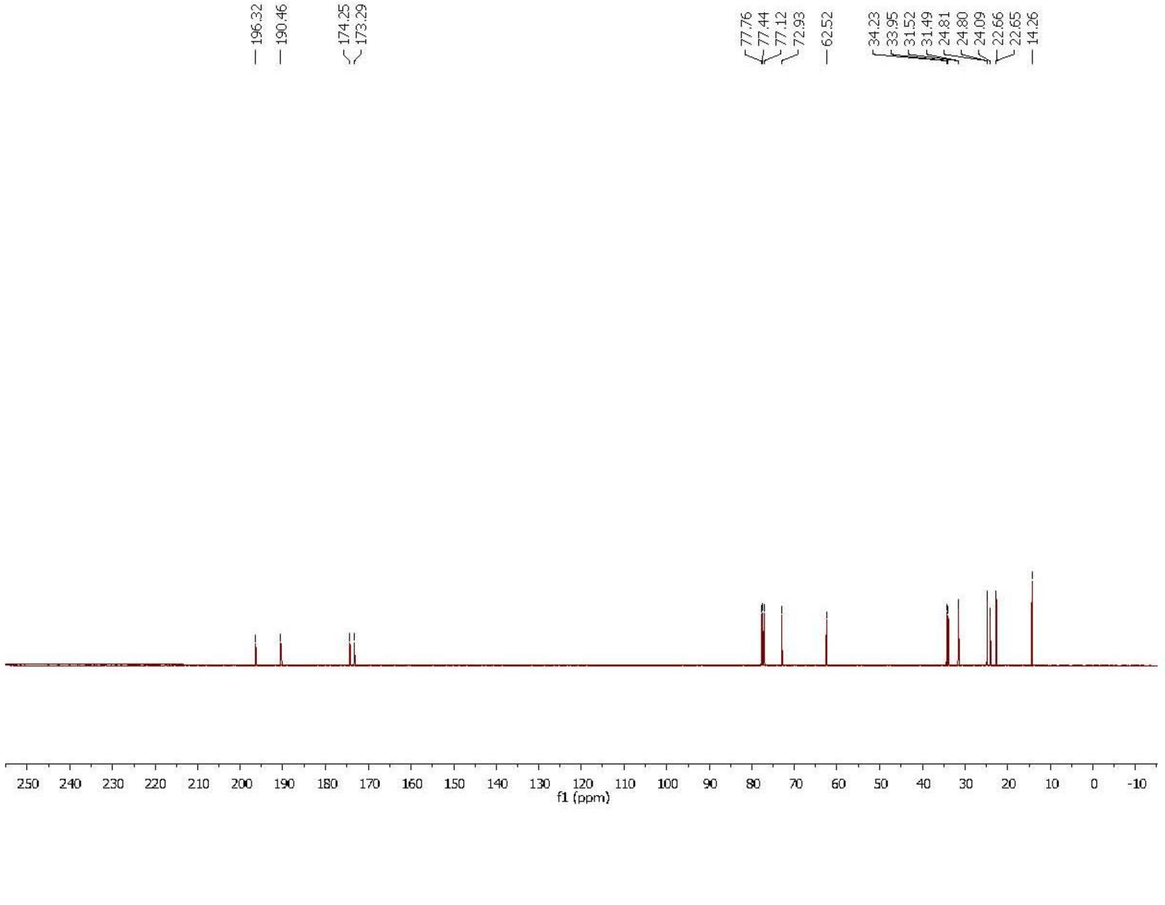

Figure S17. ^{1}H NMR (top) and ^{13}C NMR (bottom) spectra of 6-methyl-3,4-dioxoheptane-1,2-diyl diacetate (23).

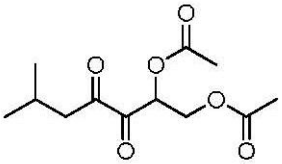

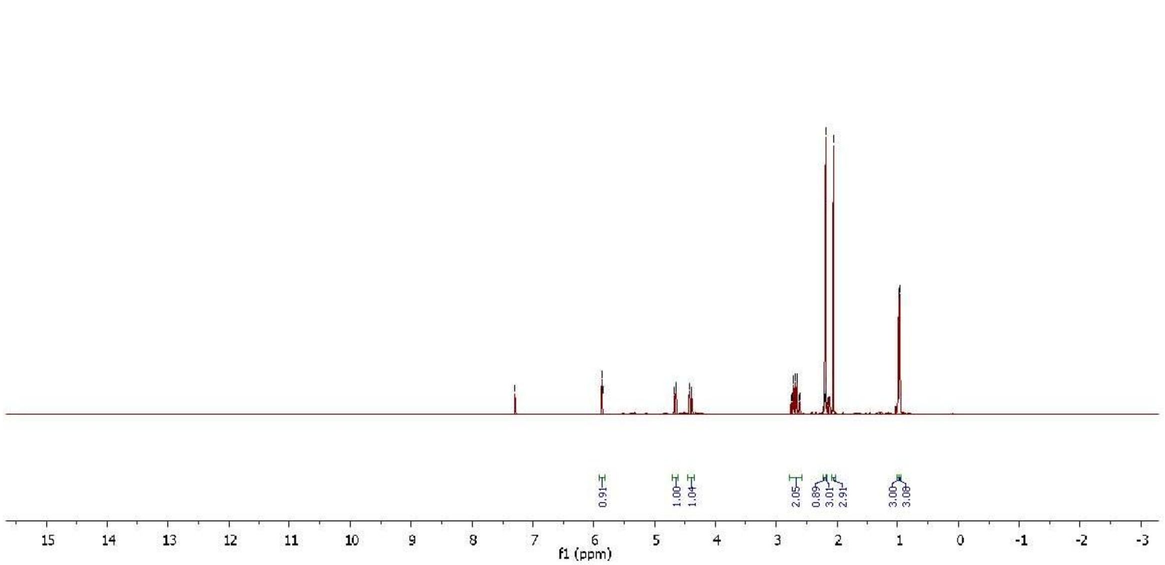

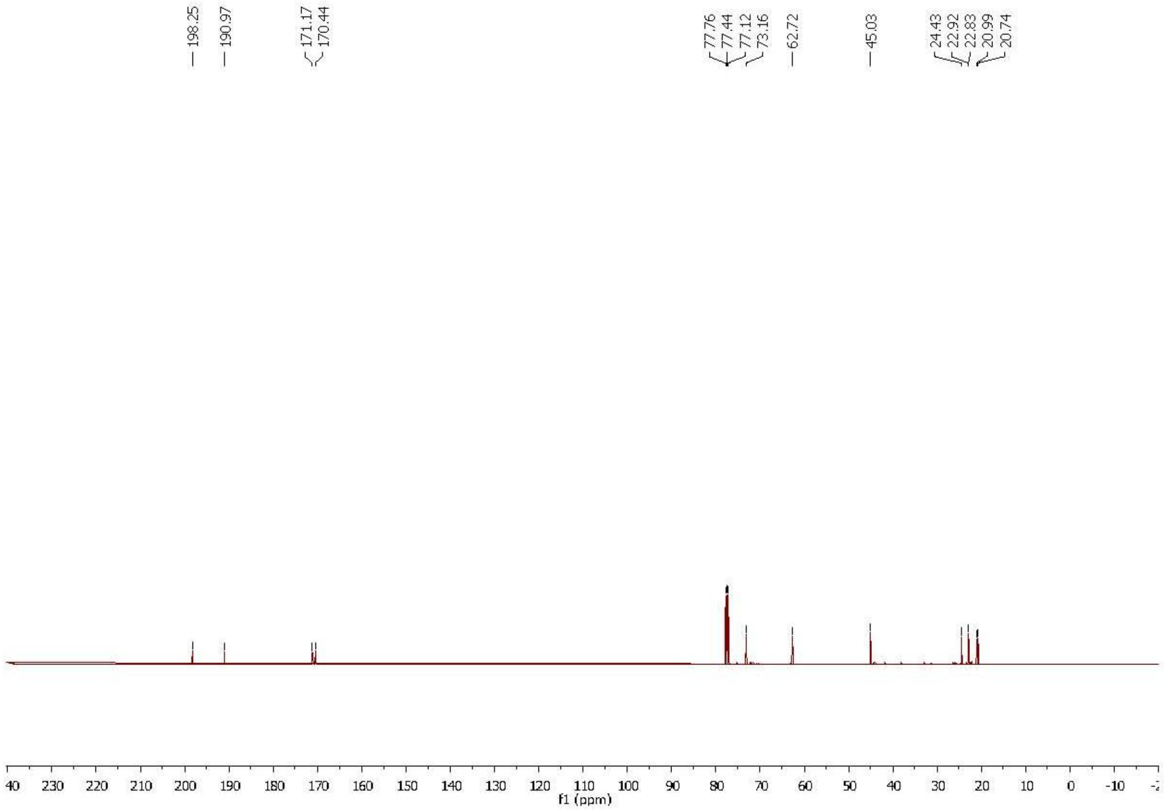

Figure S18. ^{1}H NMR (top) and ^{13}C NMR (bottom) spectra of 6-methyl-3,4-dioxoheptane-1,2-diyl dibutyrate (24).

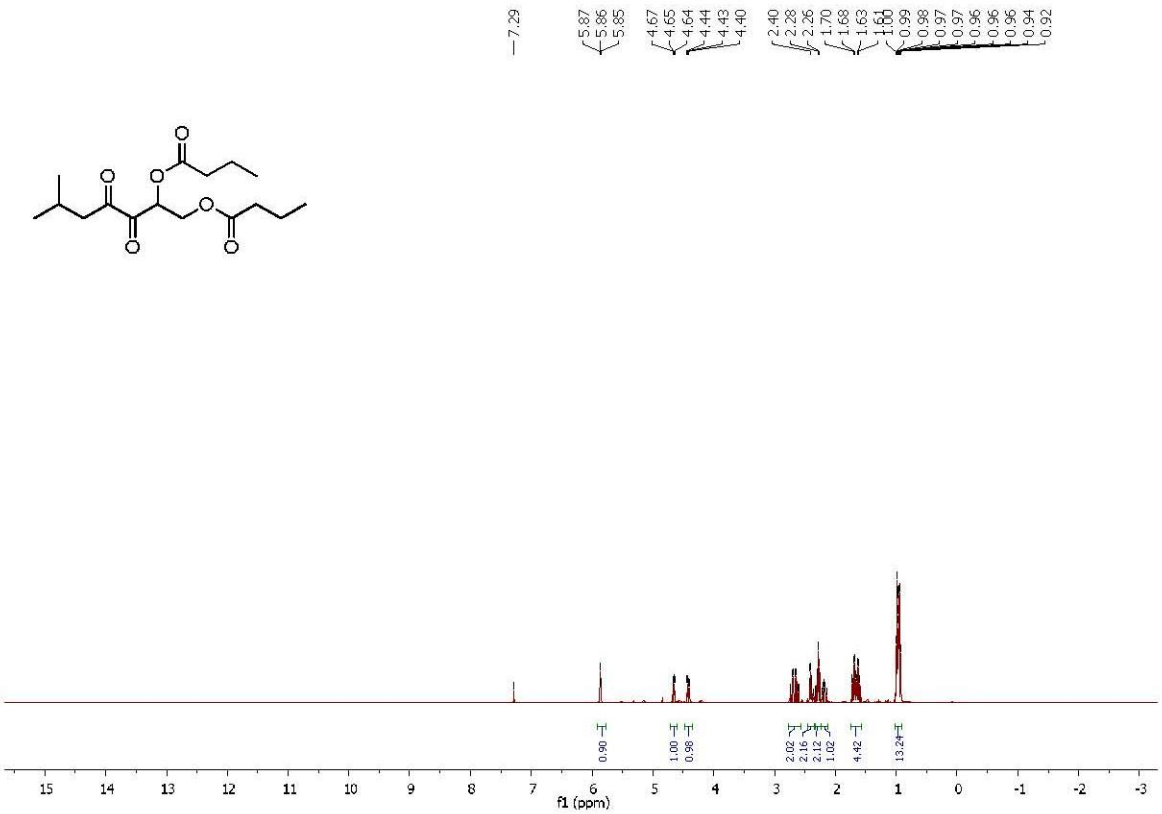

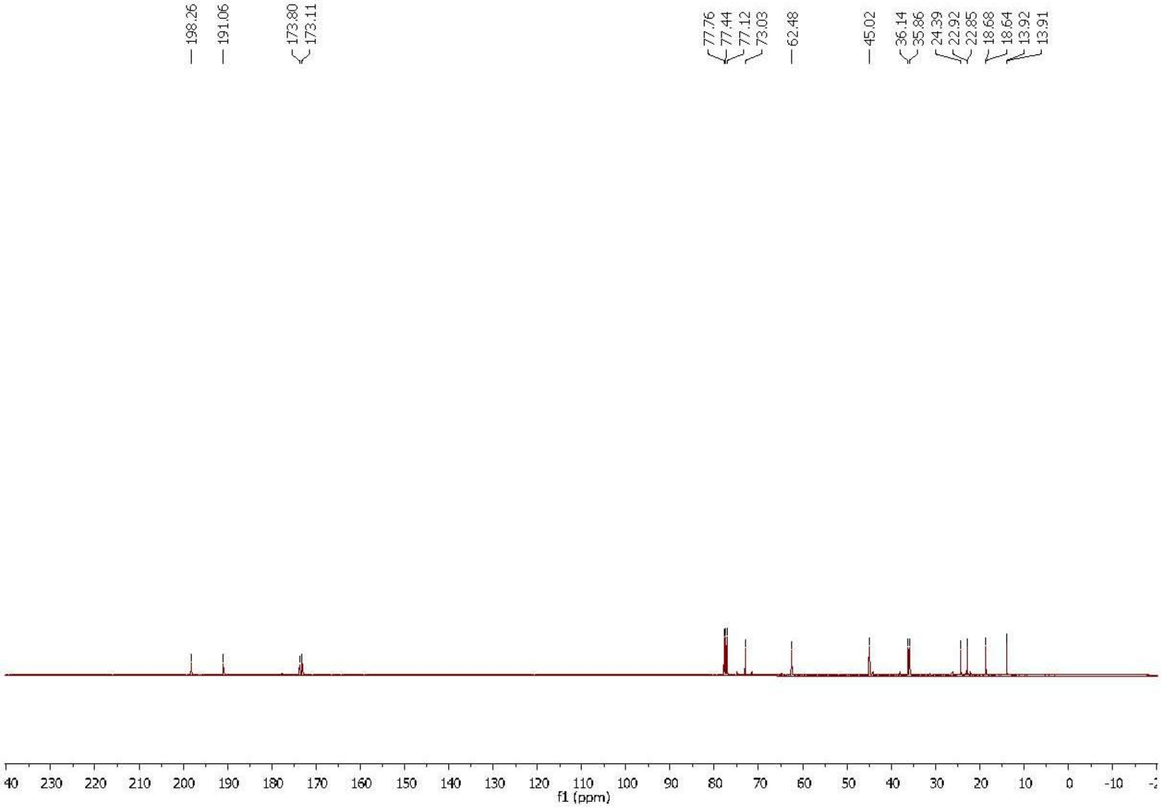

Figure S19. ^{1}H NMR (top) and ^{13}C NMR (bottom) spectra of 6-methyl-3,4-dioxoheptane-1,2-diyl dipentanoate (25).

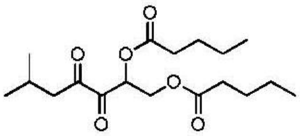

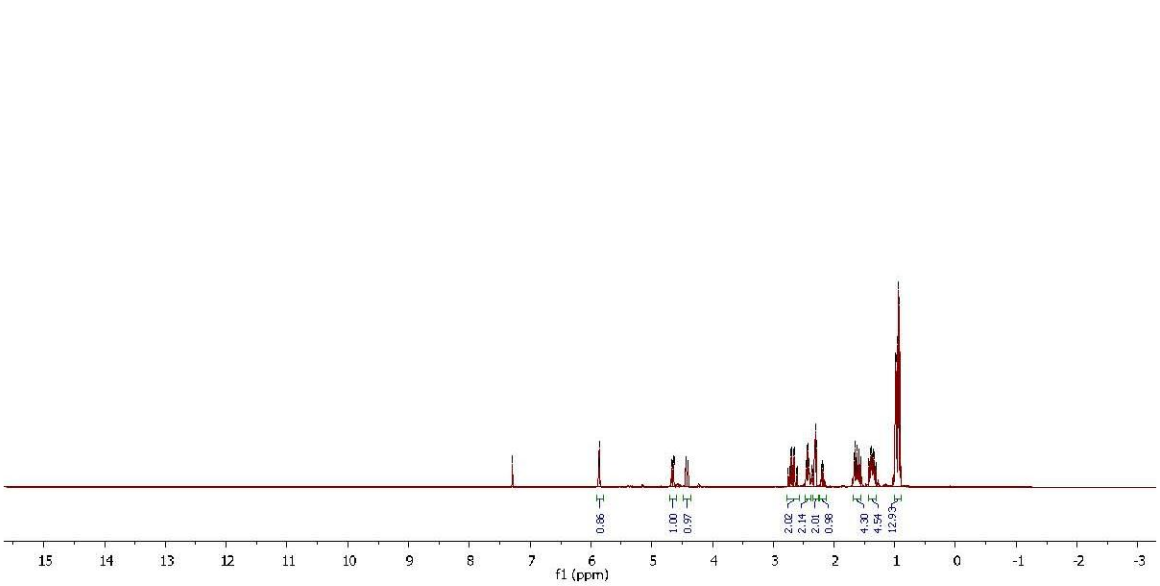

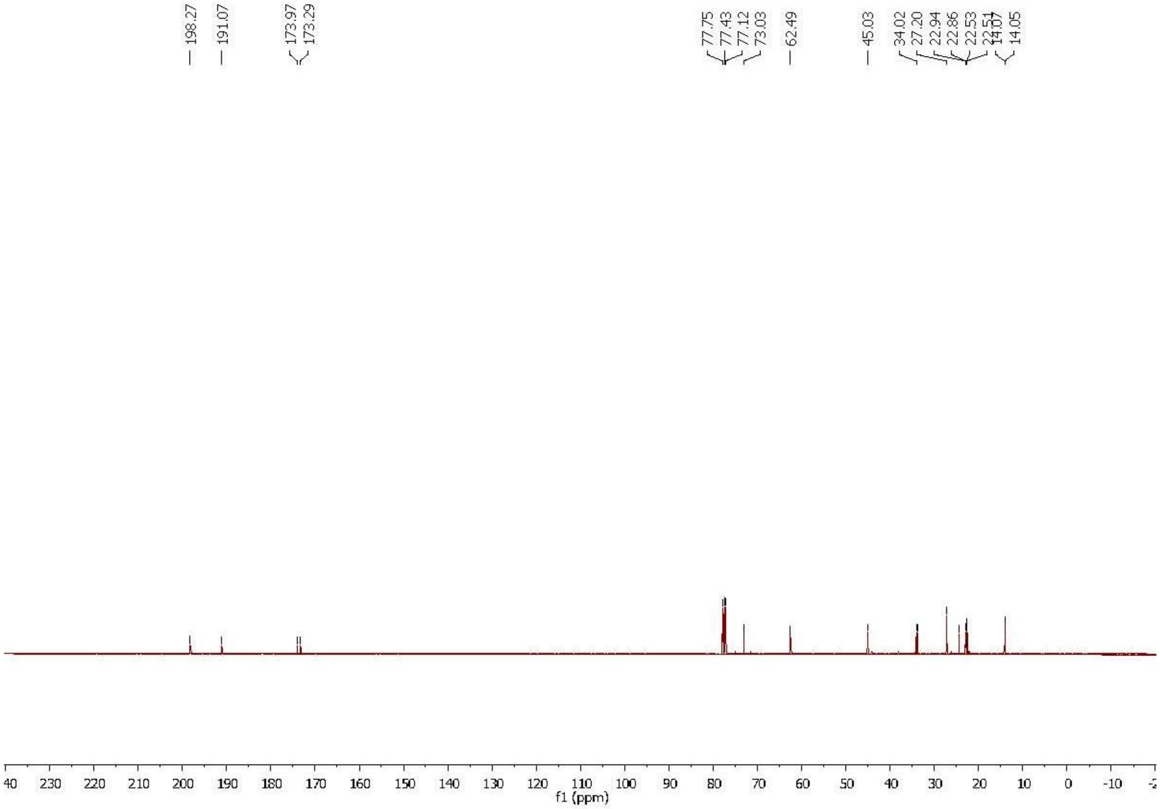

Figure S20. ^{1}H NMR (top) and ^{13}C NMR (bottom) spectra of 6-methyl-3,4-dioxoheptane-1,2-diyl dihexanoate (26).

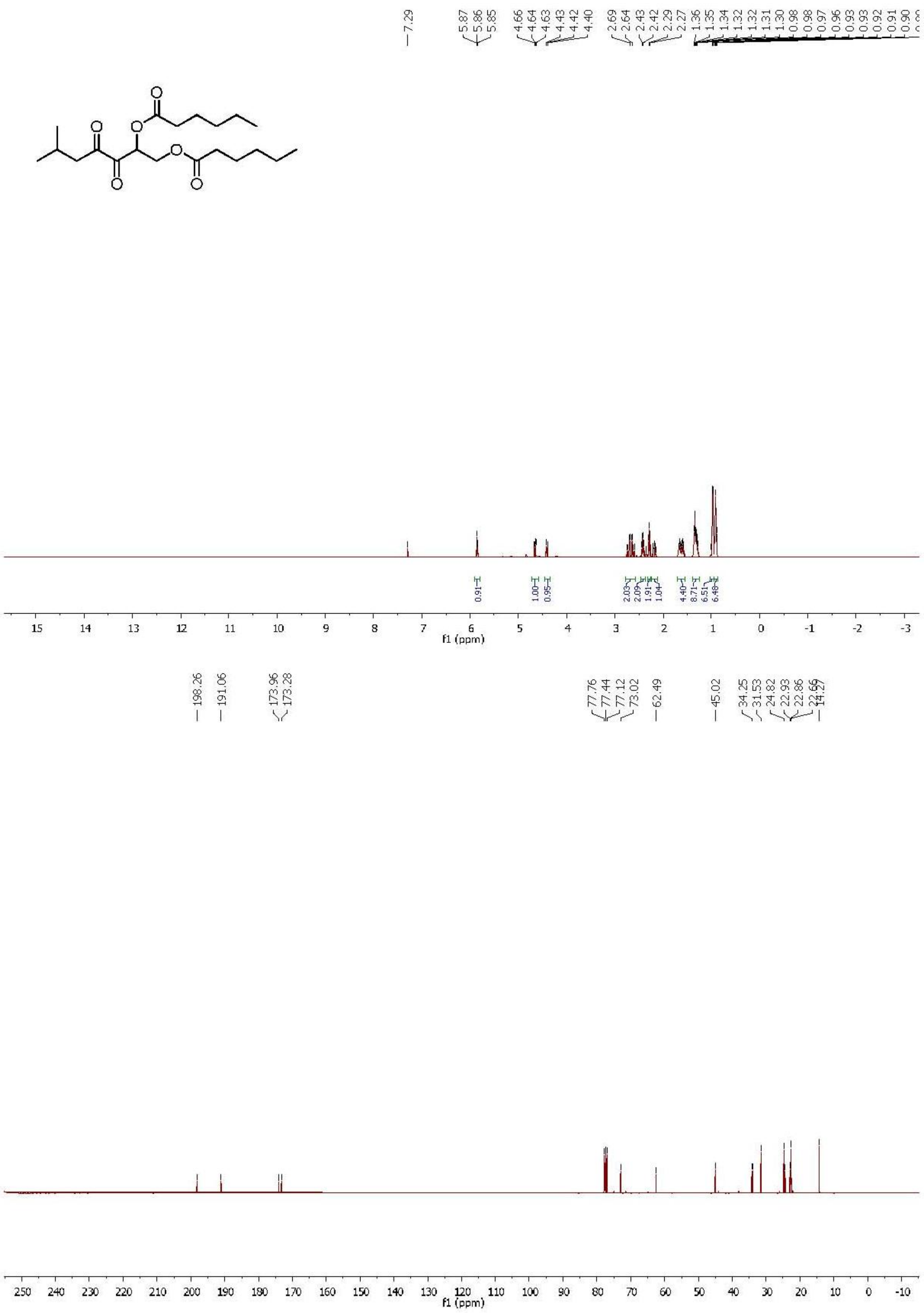

Figure S21. ^{1}H NMR (top) and ^{13}C NMR (bottom) spectra of 3,4-dioxodecane-1,2-diyl diacetate (27).

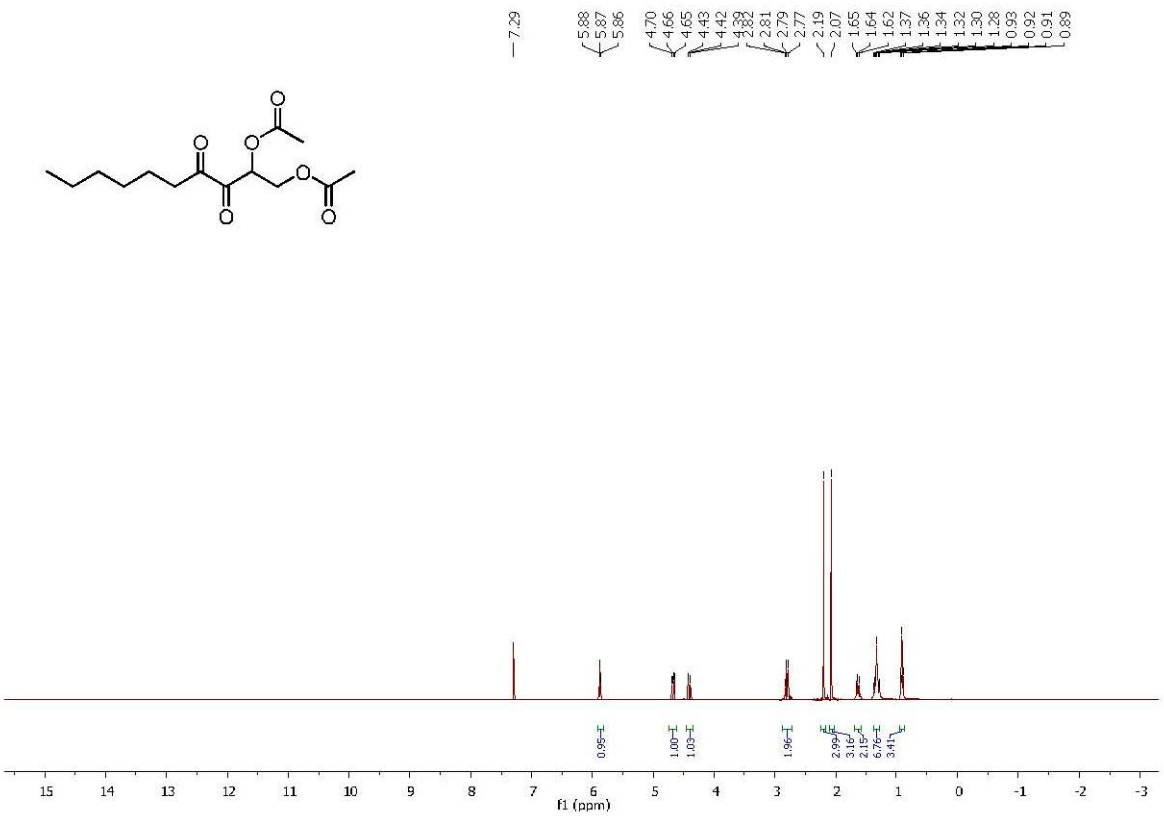

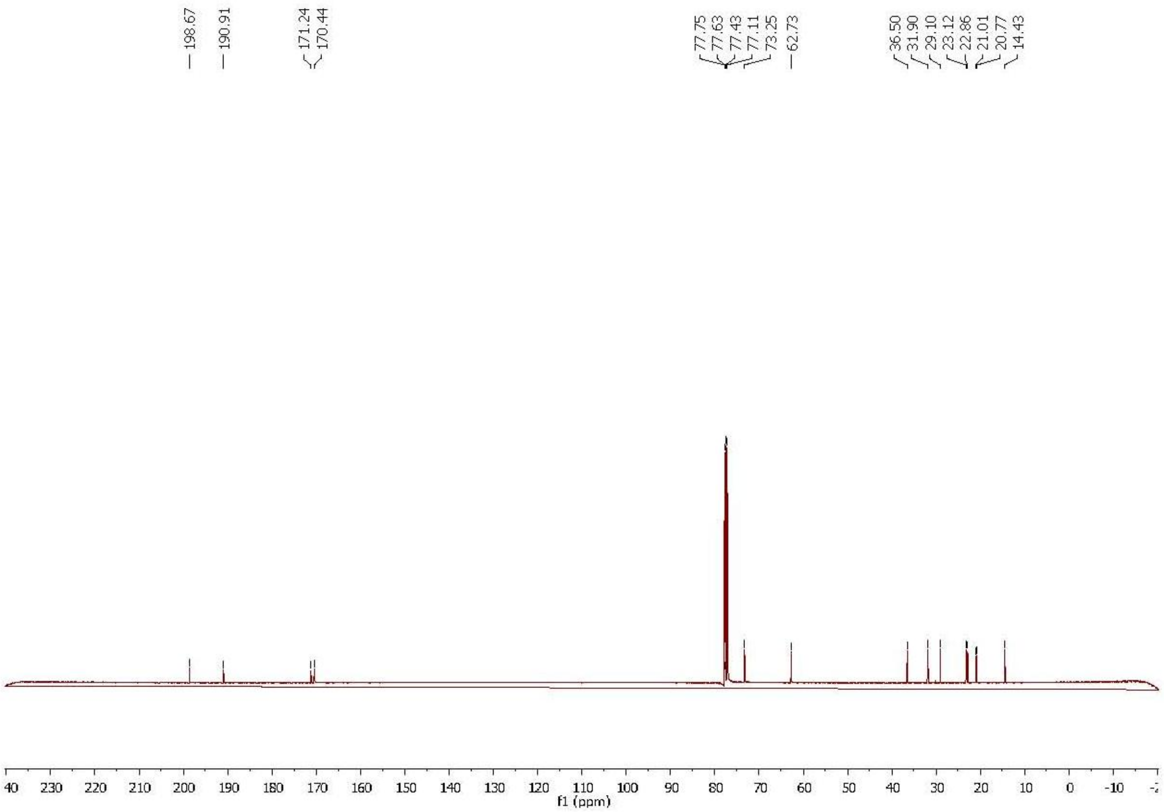

Figure S22. ^{1}H NMR (top) and ^{13}C NMR (bottom) spectra of 3,4-dioxodecane-1,2-diyl dibutyrate (28).

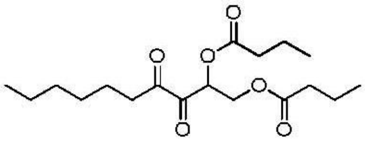

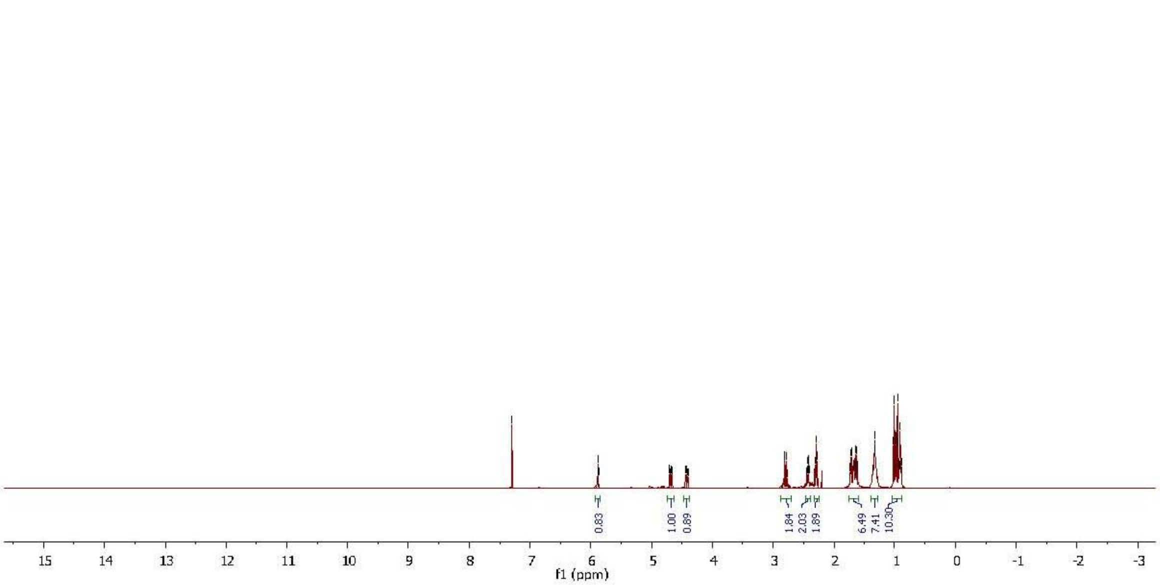

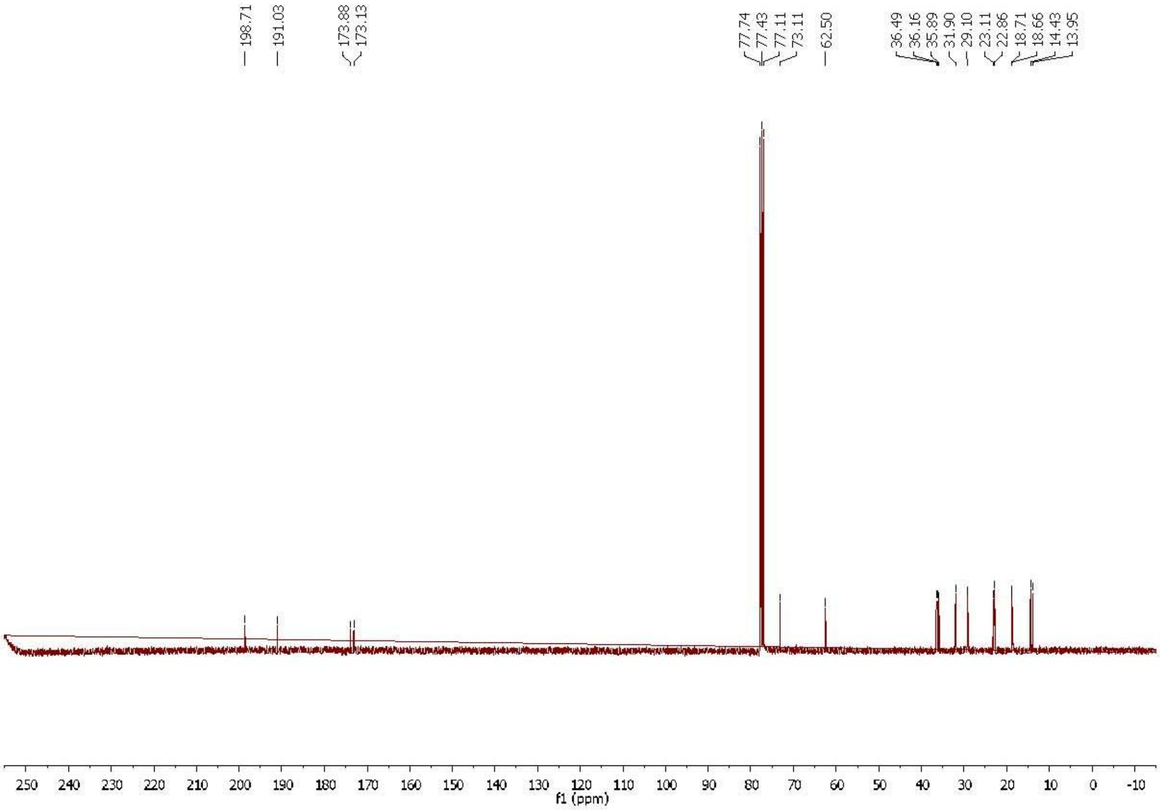

Figure S23. ^{1}H NMR (top) and ^{13}C NMR (bottom) spectra of 3,4-dioxodecane-1,2-diyl dipentanoate (29).

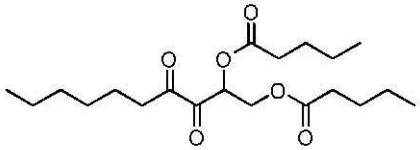

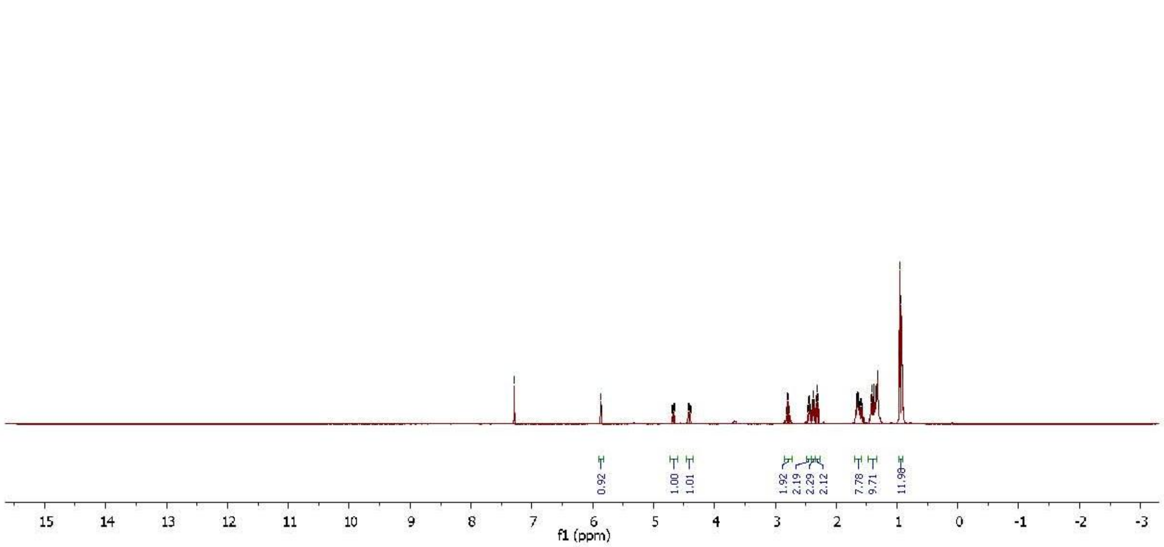

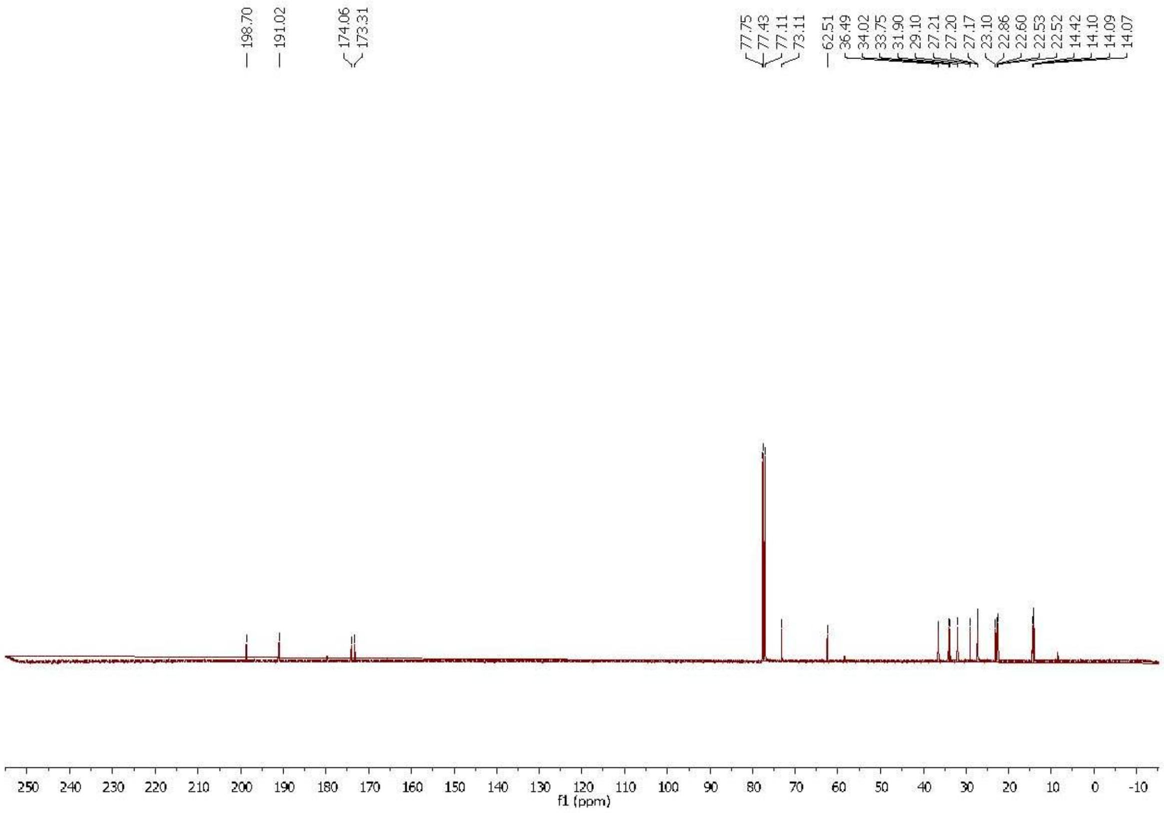

Figure S24. ^{1}H NMR (top) and ^{13}C NMR (bottom) spectra of 3,4-dioxodecane-1,2-diyl dihexanoate (30).

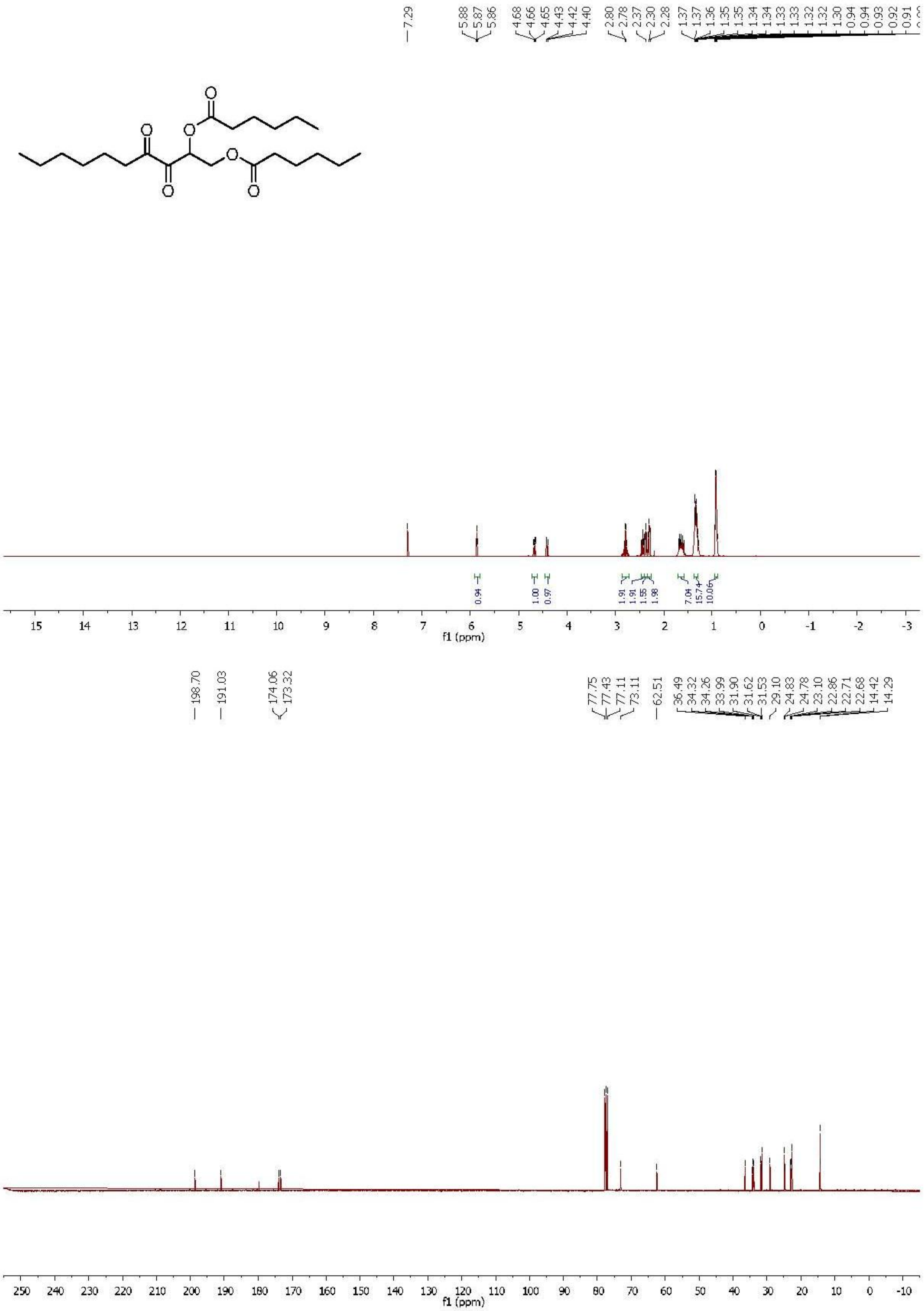

References

1. Smith, J.A.I.; Wang, J.; Nguyen-Mau, S.-M.; Lee, V.; Sintim, H.O. Biological screening of a diverse set of AI-2 analogues in *Vibrio harveyi* suggests that receptors which are involved in synergistic agonism of AI-2 and analogues are promiscuous. *Chem. Commun. (Camb.)* **2009**, *45*, 7033–7035.

© 2012 by the authors; licensee MDPI, Basel, Switzerland. This article is an open access article distributed under the terms and conditions of the Creative Commons Attribution license (http://creativecommons.org/licenses/by/3.0/).
